# Transcriptome analysis reveals TMPRSS6 isoforms with distinct functionalities

**DOI:** 10.1111/jcmm.13562

**Published:** 2018-02-14

**Authors:** Sébastien P. Dion, François Béliveau, Antoine Désilets, Mariana Gabriela Ghinet, Richard Leduc

**Affiliations:** ^1^ Department of Pharmacology‐Physiology Faculty of Medicine and Health Sciences Université de Sherbrooke Sherbrooke QC Canada; ^2^ Institut de Pharmacologie de Sherbrooke Faculty of Medicine and Health Sciences Université de Sherbrooke Sherbrooke QC Canada

**Keywords:** hepcidin, HJV, IRIDA, iron regulation, matriptase‐2, serine protease, TMPRSS6

## Abstract

TMPRSS6 (matriptase‐2) is a type II transmembrane serine protease involved in iron homoeostasis. At the cell surface of hepatocytes, TMPRSS6 cleaves haemojuvelin (HJV) and regulates the BMP/SMAD signalling pathway leading to production of hepcidin, a key regulator of iron absorption. Although four *TMPRSS6* human isoforms and three mice *Tmprss6* isoforms are annotated in databases (Ensembl and RefSeq), their relative expression or activity has not been studied. Analyses of RNA‐seq data and RT‐PCR from human tissues reveal that *TMPRSS6* isoform 1 (*TMPRSS6*‐*1*) and *3* are mostly expressed in human testis while *TMPRSS6*‐*2* and *TMPRSS6*‐4 are the main transcripts expressed in human liver, testis and pituitary. Furthermore, we confirm the existence and analyse the relative expression of three annotated mice *Tmprss6* isoforms. Using heterologous expression in HEK293 and Hep3B cells, we show that all human TMPRSS6 isoforms reach the cell surface but only TMPRSS6‐1 undergoes internalization. Moreover, truncated TMPRSS6‐3 or catalytically altered TMPRSS6‐4 interact with HJV and prevent its cleavage by TMPRSS6‐2, suggesting their potential role as dominant negative isoforms. Taken together, our results highlight the importance of understanding the precise function of each TMPRSS6 isoforms both in human and in mouse.

## 1 INTRODUCTION

Human type II transmembrane serine proteases (TTSPs) are a family of enzymes that consists of 17 members sharing common structural features and are considered key players of cell surface proteolysis.^1^ Many TTSPs have been associated with biological processes and pathologies, which positions them as attractive therapeutic targets for diseases, such as viral infection and cancers.^2^, ^3^, ^4^

TMPRSS6 (also known as matriptase‐2) was first identified from foetal liver cDNA analysis,^5^ and genetic mutations were later associated with iron‐refractory iron deficiency anaemia (IRIDA).^6^ Since then, over 40 *TMPRSS6* mutations related to IRIDA have been identified.^7^, ^8^, ^9^, ^10^ Mechanistically, TMPRSS6 acts as a negative regulator of the *HAMP* gene, which codes for a circulatory hormone called hepcidin that lowers iron levels through ferroportin internalization.^11^, ^12^ At the cell surface, TMPRSS6 cleaves haemojuvelin (HJV), a bone morphogenetic protein (BMP) coreceptor that regulates the BMP/SMAD signalling pathway leading to *HAMP* expression.^13^, ^14^ Some *TMPRSS6* mutations have been shown to prevent HJV cleavage either directly by altering TMPRSS6 enzymatic activity or by preventing the protease from reaching the cell surface.^15^, ^16^ Conceivably, because TMPRSS6 inhibition could potentially lower circulatory iron levels by elevating hepcidin levels, TMPRSS6 has become an appealing therapeutic target for diseases characterized by iron overload.^17^, ^18^ As proof of this, genetic knockdown of *TMPRSS6* in mouse models of β‐thalassaemia and haemochromatosis reduces iron overload‐related characteristics and symptoms.^19^, ^20^

Velasco et al.^5^ first described TMPRSS6 as an 802‐amino acid (aa) protein mostly expressed in the liver, but isoforms of various lengths have been annotated in different databases with some discrepancies. In fact, it is the 802aa‐form (*TMPRSS6* isoform 2, according to the UniProt Consortium nomenclature^21^) that has been most commonly used.^5^, ^16^, ^22^, ^23^, ^24^, ^25^ However, other groups, including ours, have focussed on *TMPRSS6‐1* (811 aa), which is known as the “*TMPRSS6* canonical isoform”.^13^, ^15^, ^26^, ^27^, ^28^, ^29^ The difference between the two isoforms is the expression of *TMPRSS6‐1* coding exon 1, which encodes for residues 1‐9 in the N‐terminal, cytoplasmic portion of the protein.^30^ Although two other well‐supported *TMPRSS6* isoforms are annotated in UniProt^21^ and Ensembl^30^ databases, neither their relative expression nor their respective functionalities have been studied. In mice, *Tmprss6* isoforms annotation is not constant in the different databases but three distinct coding transcripts have been annotated in NCBI Reference Sequence Database (RefSeq).^31^

Using transcriptome analysis and heterologous expression, we confirm the existence and relative abundance of the different isoforms in both species. More importantly, we found revealing differences in the functionality of human *TMPRSS6* isoforms. Because TMPRSS6 is such a critical player in iron regulation and a promising therapeutic target, we wanted to highlight the importance of knowing precisely which isoforms are expressed in human tissues and to characterize the distinct functional properties of these isoforms.

## 2 MATERIALS AND METHODS

### 2.1 Cells, antibodies and reagents

HEK293 and Hep3B cells were purchased from American Type Culture Collection (Manassas, VA). HEK293 and Hep3B cells were respectively maintained in high glucose Dulbecco's Modified Eagle's Medium (DMEM) or Eagle's Minimum Essential Medium (EMEM) both containing 10% foetal bovine serum, 2 mmol/L l‐glutamine, 100 IU/mL penicillin and 100 μg/mL streptomycin or serum‐free media HCELL‐100 (WISENT, St‐Bruno, Canada). Poly‐l‐lysine‐coated coverslips were from Corning (Bedford, MA). Restriction enzymes XhoI and KpnI‐HF were from New England Biolabs (Ipswich, MA). Anti‐V5, HRP and FITC‐linked Anti‐V5 monoclonal antibodies (mAb) were from Invitrogen (Waltham, MA). HRP‐linked anti‐GAPDH rabbit mAb was from Cell Signaling Technology (Danvers, MA). Goat polyclonal anti‐Haemojuvelin antibody and t‐butoxycarbonyl‐Gln‐Ala‐Arg‐7‐amino‐4‐methylcoumarin (Boc‐QAR‐AMC) were purchased from R&D Systems (Minneapolis, MN). Lipofectamine 3000 was from Invitrogen (Carlsbad, CA). Centrifugal filters were from Merck Millipore (Cork, Ireland). Lysis buffer (1% Triton, 50 mmol/L Tris, 150 mmol/L NaCl, 5 mmol/L EDTA) was supplemented with protease inhibitor from Roche (Mannhem, Germany). Protein A/G PLUS‐agarose beads were from Santa‐Cruz Biotechnology (Dallas, TX). C57BL/6 WT mice were from Charles River (Montréal, Canada). Ketamine was from Vétoquinol (Lavaltrie, Canada). Xylazine was from Bimeda (Cambridge, Canada). Surflo Winged Infusion Set was from Terumo (Tokyo, Japan). Liver Perfusion Medium, Liver Digest Medium, Hepatocyte Wash Medium and William's E Medium (supplemented with Primary Hepatocyte Thawing and Plating Supplements) were from Life Technologies (Grand Island, NY). Percoll was from GE Healthcare (Uppsala, Sweden). TRIzol was from Life Technologies (Carlsbad, CA). Liver cDNA pool was from (BioChain, Newark, CA). ProteoExtract Native Membrane Extraction Kit was from Millipore (Darmstadt, Germany).

### 2.2 RNA‐sequencing (RNA‐seq) data analysis

Expression of *TMPRSS6* transcripts and iron‐related genes in human tissue samples (RPKM; reads per kilobase of exon per million fragments mapped) were obtained from the Genotype‐Tissue Expression (GTEx) project (release V6p).^32^ GTEx sample identification numbers (id) used for all the analysed tissues are listed in Table S1. *TMPRSS6‐3* (ENST00000442782) expression levels in human cell lines were obtained from RNA‐seq analysis of The Human Protein Atlas version 16.1.^33^ Mouse liver samples were retrieved from the European Nucleotide Archive (ENA; http://www.ebi.ac.uk/ena). The accession numbers are listed in Table S2. The obtained reads from RNA‐seq datasets were aligned to the mouse reference genome GRCm38/mm10 performed with HISAT2 v2.03.^34^ Genes and transcript RPKM expression values were calculated with Cufflinks v2.2.1.0^35^ using the modified annotated transcriptome from ENSEMBL (ftp://ftp.ensembl.org/pub/release-75/gtf/mus_musculus), including *Tmprss6* isoform 2 (NM_001355601.1) and X2 (XM_006521417.2) (Table S3), as a reference.

### 2.3 Mouse primary hepatocytes

Mouse primary hepatocytes were obtained from C57BL/6 mice. Animals were anesthetized with ketamine/xylazine. The liver was perfused with 70 mL of Liver Perfusion Medium prior to the perfusion with Liver Digest Medium. Liver was then cut into small pieces and dissociated in Hepatocyte Wash Medium. Viable cells were plated in 6‐well plates and were washed with PBS 24 hours post‐plating. Cells were treated with TRIzol reagent to further isolate and analyse RNA. The use of animals in the context of this project was approved by the Université de Sherbrooke Animal Ethic Committee. Additional details are provided in the supplemental data.

### 2.4 RT‐PCR

Human liver cDNA was from Applied Biosystems (Foster City, CA). Human liver RNA pool from 5 healthy donors was from BioChain (Newark, CA). XpressRef Universal Total RNA (total human RNA) was from Qiagen (Germantown, MD). Mouse primary hepatocytes RNA were extracted from a 6‐well plate covered by adherent cells using TRIzol with chloroform, following the manufacturer's protocol. The aqueous layer was recovered, mixed with one volume of 70% ethanol and applied directly to an RNeasy Mini Kit column (Qiagen, Germantown, MD). DNAse treatment on the column and total RNA recovery were performed as per the manufacturer's protocol. RNA quality and presence of contaminating genomic DNA were verified as previously described.^34^ RNA integrity was assessed with an Agilent 2100 Bioanalyzer (Agilent Technologies). Reverse transcription on RNA samples was performed on 1 μg total RNA with Transcriptor reverse transcriptase, random hexamers, dNTPs (Roche Diagnostics, Laval, Canada) and 10 units of RNAseOUT (Life Technologies, Carlsbad, CA) following the manufacturer's protocol in a total volume of 10 μL. PCR reactions on human liver cDNA were prepared using 0.5 ng cDNA and AmpliTAQ Gold 360 Master Mix (Applied Biosystems, Carlsbad, CA), and 0.5 μmol/L of each appropriate primer. PCR reactions on cDNA from human liver pool RNA and XpressRef Universal Total RNA were prepared using 1 ng cDNA and Q5 Hot Start High‐Fidelity 2X Master Mix (New England Biolabs, Ipswitch, MA) with 0.5 μmol/L of each appropriate primer. For the human primer details and list, see Figure S1 and Table S4 and for the mice primer list see Figure S2 and Table S5.

### 2.5 Plasmid construction

The cDNAs encoding TMPRSS6‐1 and HJV were obtained and cloned as previously described.^26^ TMPRSS6‐2 construct was obtained using the QuikChange site‐directed mutagenesis kit (Agilent Technologies, Santa Clara, CA). TMPRSS6‐3 and TMPRSS6‐4 constructs were obtained by inserting a synthetic double‐stranded DNA block coding for the isoform sequence into a modified form of pcDNA6/V5‐His (Invitrogen) that was previously described.^26^ Additional details are provided in the supplemental data.

### 2.6 Immunofluorescence

Cells were seeded on poly‐l‐lysine‐coated coverslips. Cells were transfected with appropriate plasmids. Twenty‐four hours later, cell surface TMPRSS6 was labelled for 1 hour at 4°C. Cells were washed and incubated at 37°C for 15 or 30 minutes. Cells were then prepared as previously described.^35^ Cells were examined on an inverted spectral scanning confocal microscope FV1000 (Olympus, Tokyo, Japan). Additional details are provided in the supplemental data.

### 2.7 Expression and detection of TMPRSS6

Cells were transfected with TMPRSS6 isoform cDNAs performed with Lipofectamine 3000 in 6‐well plates. Twenty‐four hours later, the cell media were replaced with HCELL‐100 media for 24 hours. Cell media were collected and concentrated, and cells were lysed. Samples were loaded on 12% SDS‐PAGE and analysed by immunoblotting. Additional details are provided in the supplemental data.

### 2.8 Proteolytic activity measurements in the cell media

At 24 hours post‐transfection, the cell media were replaced with HCELL‐100 media for another 24 hours. The media were collected, and activity was measured by the release of fluorescence from Boc‐QAR‐AMC cleavage.^26^ Additional details are provided in the supplemental data.

### 2.9 Membrane isolation and proteolytic activity measurements of isolated membrane fractions

Hep3B cells were transfected with TMPRSS6 isoform cDNAs performed with Lipofectamine 3000 in 10 cm culture dishes. Membranes were then isolated using ProteoExtract Native Membrane Extraction Kit as previously described.^36^ The activity in the membrane fractions was measured using Boc‐QAR‐AMC fluorogenic substrate in assay buffer as previously described.^37^ Samples were loaded on 12% SDS‐PAGE and analysed by immunoblotting.

### 2.10 Statistical analysis

Statistical analyses were conducted using GraphPad Prism version 7.0c (GraphPad Software, La Jolla, CA). Outliers were removed using the ROUT method (Q = 1%). One‐sample *t* test analysis (hypothetical mean fixed at 1) was used to compare the activity of isoforms relative to mock transfected cells (fold induction). *P* values <.05 were considered statistically significant (†). Normality was assessed using the D'Agostino‐Pearson omnibus normality test before using non‐parametric Kruskal‐Wallis test to compare the activity between the isoforms. *P* values <.05 were considered statistically significant (*).

### 2.11 Interaction between TMPRSS6 and HJV

Cells were cotransfected with the TMPRSS6 isoform cDNA and HJV transcript variant a. At 24 hours post‐transfection, cells were washed and harvested on ice in lysis buffer. Protein samples were immunoprecipitated with an anti‐V5 antibody and Protein A/G PLUS‐agarose beads for 24 hours at 4°C. Immunoprecipitated proteins were loaded on 10% or 12% SDS‐polyacrylamide gels and analysed by immunoblotting. Additional details are provided in the supplemental data.

### 2.12 HJV processing by TMPRSS6

Cells were cotransfected with one or two TMPRSS6 isoforms cDNA and HJV transcript variant a. At 24 hours post‐transfection, cell media were replaced with HCELL‐100 for another 24 hours then collected and concentrated. The cells were lysed and samples were loaded on 12% SDS‐polyacrylamide gels and analysed with immunoblotting. Additional details are provided in the supplemental data.

## 3 RESULTS

### 3.1 Expression of *TMPRSS6* transcripts in human

**Figure 1:**
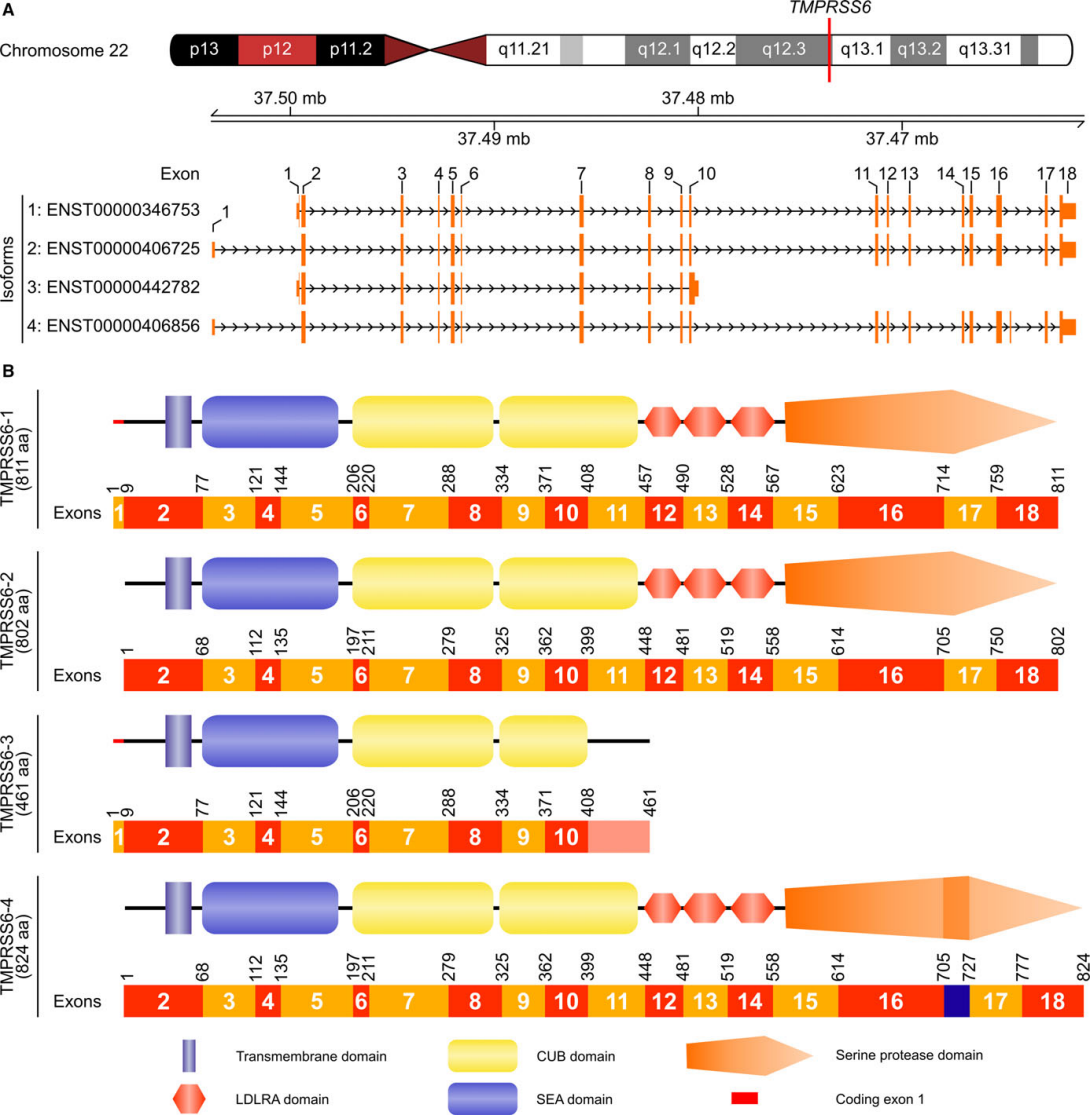
Human *TMPRSS6* isoforms. (A) Schematic representation of human chromosome 22 including *TMPRSS6* locus and *TMPRSS6* transcripts (Ensembl annotation, GRCh37). Non‐coding regions are displayed smaller than coding regions. (B) Representation of TMPRSS6 isoforms and corresponding coding exons. Schematic representation of TMPRSS6 is adapted from a previous article published by our group^26^

The human *TMPRSS6* gene is located on chromosome 22 (22q12.3) and is expressed as 7 known different transcripts (Ensembl database), but only 4 of them have a well‐supported annotation and are predicted to be expressed as proteins (Figure 1A).^30^ These transcripts encode 4 *TMPRSS6* isoforms that lead to the production of different proteins (Figure 1B). To facilitate reading, a 1‐4 numbering nomenclature (according to the UniProt Consortium)^21^ is used in this study and has been linked to the Ensembl transcript annotation. *TMPRSS6‐1* (ENST00000346753, RefSeq NM_153609, 811 aa) has 18 coding exons and is considered the canonical variant of *TMPRSS6*.^21^ *TMPRSS6‐2* (ENST00000406725, RefSeq NM_001289001, 802 aa) also has 18 exons but exon 1 is different and non‐coding. Residues 1‐9 from the N‐terminal, cytoplasmic tail, are present in isoform 1 but absent in isoform 2. *TMPRSS6‐3* has two different annotations, including one coding for a protein of 461 aa (ENST00000442782) that expresses the first 9 coding exons of *TMPRSS6‐1* but has an alternatively spliced form of exon 10 that drives the expression of a truncated isoform in the second CUB domain (Ensembl annotation).^30^ The second annotation for *TMPRSS6‐3* describes a protein of 452 aa (UCSC annotation uc003aqu.3) that has the same non‐coding exon 1 than *TMPRSS6‐2*. Finally, *TMPRSS6‐4* (ENST00000406856, RefSeq NM_001289000, 824 aa) also possesses the same non‐coding exon 1 than *TMPRSS6‐2* and expresses an additional exon between exons 16‐17 coding for a 22‐aa insertion in the catalytic domain.

**Figure 2:**
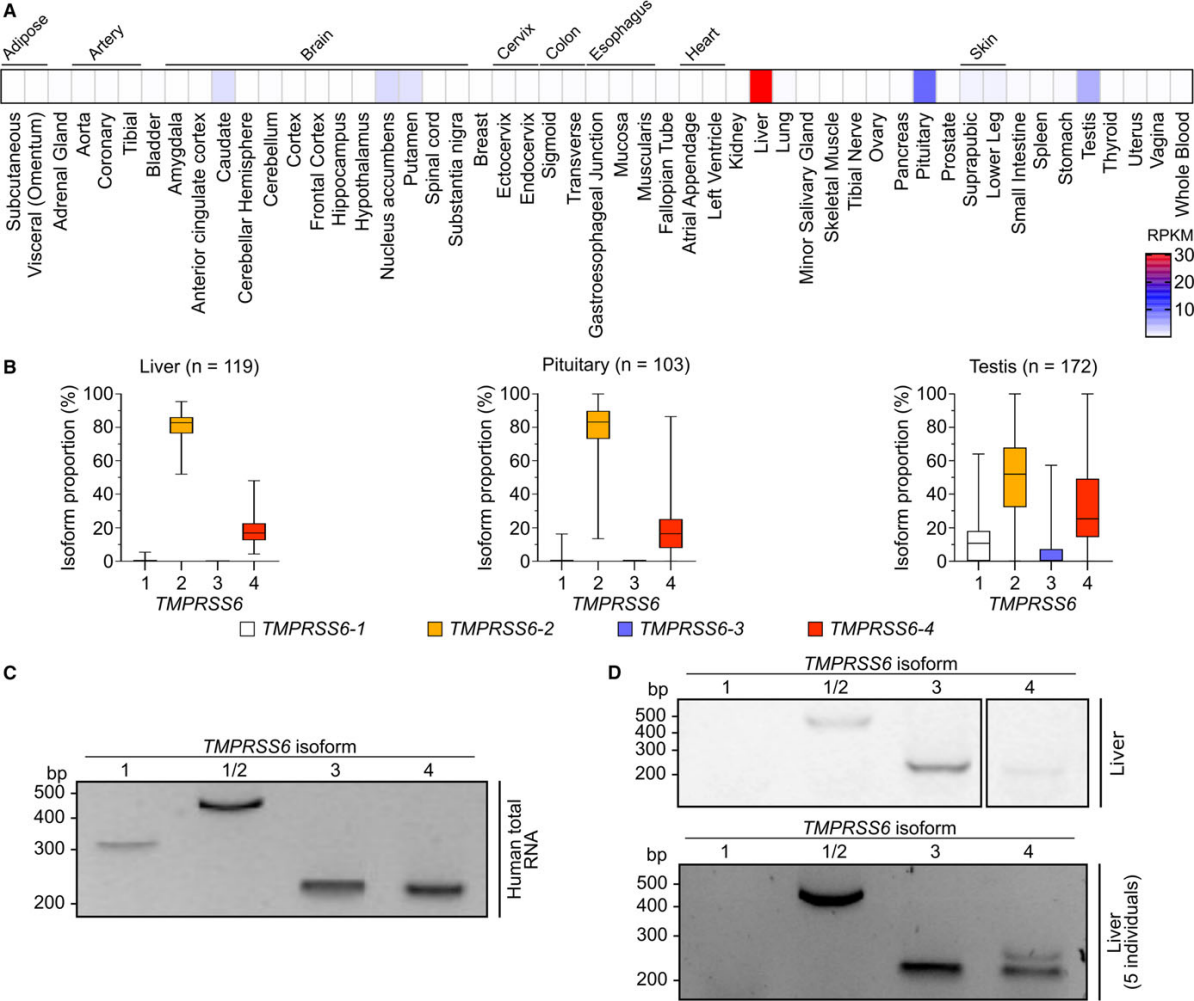
*TMPRSS6* expression in human tissues. (A) *TMPRSS6* global expression levels in healthy human tissues. Results are presented as a heat‐map of reads per kilobase of transcript per million mapped reads (RPKM) determined by RNA‐sequencing data analysis (53 tissues analysed from 544 different donors). (B) Relative *TMPRSS6* isoform expression in healthy human liver (n = 119), pituitary (n = 103) and testis (n = 172) as assessed by RNA‐sequencing data analysis. Results are presented as proportional isoform expression (%) and are shown as boxes and whiskers plot. Box‐and‐whisker plots display quartiles and range. (C) Detection of *TMPRSS6* isoforms in a total human RNA sample as assessed by RT‐PCR. (D) Detection of *TMPRSS6* isoforms in a liver RNA sample and in a RNA pool of 5 healthy human livers as assessed by RT‐PCR

To the best of our knowledge, functional differences between *TMPRSS6* isoforms have not been characterized. In fact, research reports have routinely been using TMPRSS6‐1 or TMPRSS6‐2 in transfection experiments, while no clear function for TMPRSS6‐3 or TMPRSS6‐4 has been elucidated. To gain insight on the expression of *TMPRSS6* isoforms and their abundance in human tissues, we analysed publicly available RNA‐seq datasets of the Genotype‐Tissue Expression (GTEx) project (Figure 2A).^32^ We found that *TMPRSS6* is mainly expressed in the liver with lower levels detected in the pituitary and testis. Low levels were also detected in distinct brain regions.

We further analysed the proportion of isoforms expressed in the three main expression tissues performed with RNA‐seq data analysis (Figure 2B). Surprisingly, RNA‐seq data did not enable us to confirm expression of the canonical variant *TMPRSS6‐1* in human liver and pituitary but reveals its expression in testis (10.8%). *TMPRSS6‐2* is the isoform with the highest expression levels in all three tissues while *TMPRSS6‐3* is expressed only in the testis (6.2%). Finally, *TMPRSS6‐4* is expressed in the liver (16.9%), in the pituitary (16.5%) and in the testis (25.5%). These results show that the liver and pituitary share similar expression patterns and express mainly *TMPRSS6‐2* and *TMPRSS6‐4,* while *TMPRSS6‐1* and *TMPRSS6‐3* are enriched in the testis compared to the liver.

We next confirmed the existence of the four *TMPRSS6* isoforms performed with RT‐PCR analysis on human total RNA (Figure 2C). We also detected *TMPRSS6‐2*,* TMPRSS6‐3* and *TMPRSS6‐4* in two different human RNA liver samples (single donor and pool of 5 individuals) (Figure 2D).

Taken together, these results point to *TMPRSS6‐2* as being the main transcript in liver and *TMPRSS6‐4* as a relatively abundant while *TMPRSS6‐1* and *3* are expressed at more negligible levels.

Whether *TMPRSS6‐3* contains the same exon 1 than *TMPRSS6‐1* is unclear as 2 different annotations diverge on this point. Thus, to verify expression of exon 1 in *TMPRSS6‐3*, we analysed *TMPRSS6‐3* expression in all 56 cell lines available in The Human Protein Atlas database (Figure S3).^33^ HepG2 cells expressed the highest levels of *TMPRSS6‐3,* and we observed that elongated exon 10 is present when analysing read alignments compared to liver samples, but *TMPRSS6‐1* coding exon 1 is absent (Figure S3). This suggests that *TMPRSS6‐3* is expressed as a 452 aa protein in liver‐derived cells, in accordance with the UCSC annotation.^38^

### 3.2 Expression of *Tmprss6* transcripts in mice

**Figure 3:**
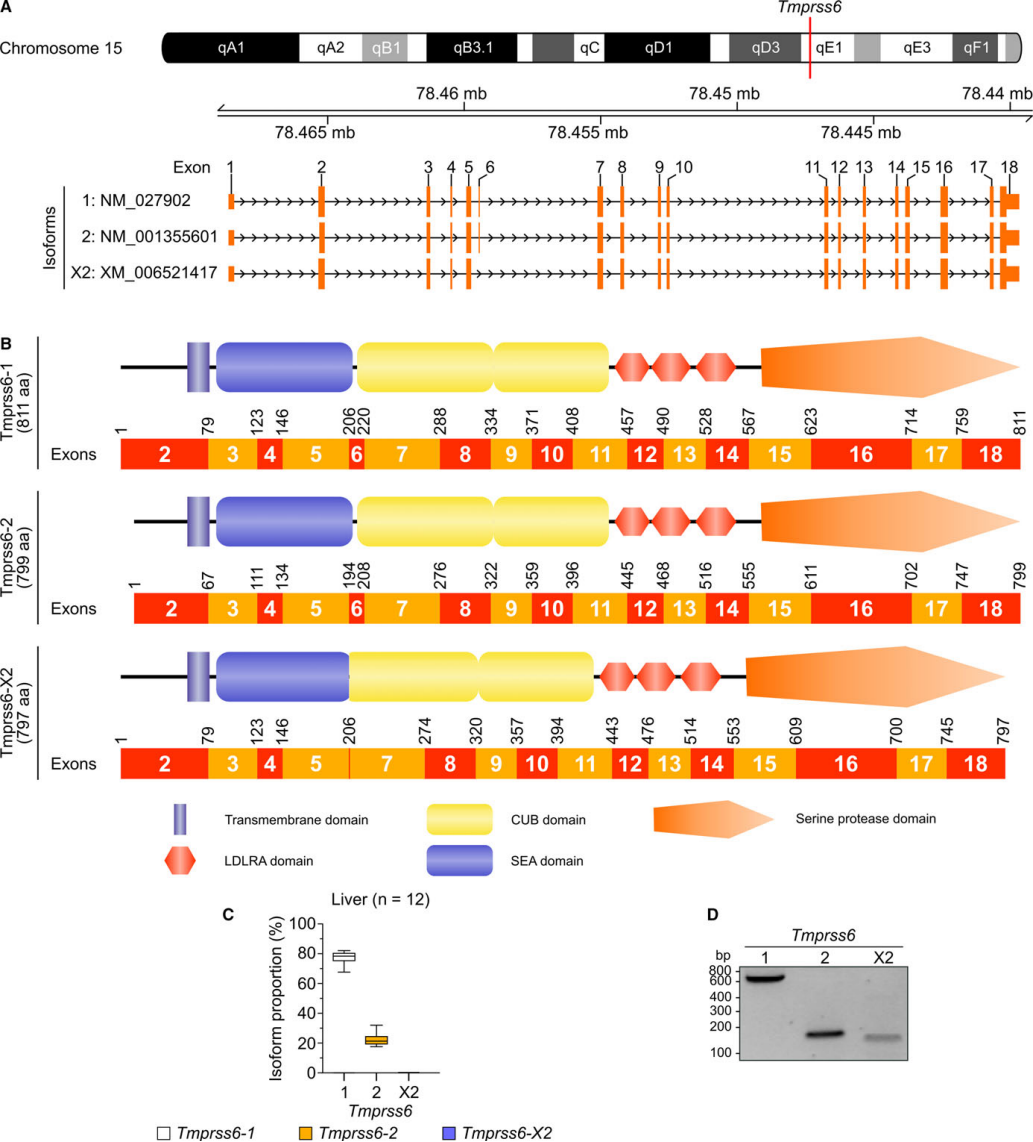
*Tmprss6* isoforms and expression in mice. (A) Schematic representation of mice chromosome 15 including *Tmprss6* locus and *Tmprss6* transcripts (RefSeq annotation). Non‐coding regions are displayed smaller than coding regions. (B) Representation of Tmprss6 isoforms and corresponding coding exons. Schematic representation of TMPRSS6 is adapted from a previous article published by our group.^26^ (C) Relative *Tmprss6* isoform expression in healthy mice livers as assessed by RNA‐sequencing data analysis (n = 12). Results are presented as proportional isoform expression (%) and are shown as boxes and whiskers plot. Box‐and‐whisker plots display quartiles and range. (D) Mice *Tmprss6* expression in mice primary hepatocytes as assessed using RT‐PCR analysis

In mice, the *Tmprss6* gene, located on chromosome 15 (15qE1), can be expressed as 3 different transcripts (Figure 3A) encoding 3 Tmprss6 isoforms (Figure 3B). The nomenclature of these isoforms relies on the RefSeq database as the Ensembl database only describes a single isoform (*Tmprss6‐1*, ENSMUST00000017086, RefSeq NM_027902).^31^ *Tmprss6‐1* and *Tmprss6‐2* (NM_001355601) are encoded by exon 2 to 18 and differ by the expression of 12 residues in their N‐terminal region leading to proteins of 811 and 799 aa, respectively. *Tmprss6‐X2* (XM_006521417) lacks exon 6, thus bringing both the SEA and the first CUB domain of the protein closer which produces a protein of 797 aa.

We analysed the expression of these isoforms within healthy mice liver samples performed with RNA‐seq data analysis. (Figure 3C). We found that *Tmprss6‐1* is the main expressed isoform (79%), with *Tmprss6‐2* in lower abundance (21%). *Tmprss6‐X2* was not detected. To confirm the presence of *Tmprss6* isoforms, we performed RT‐PCR on mice primary hepatocytes. All transcripts were detected using this technique (Figure 3D). These results confirm the existence and reveal the differential expression of annotated *Tmprss6* isoforms in mouse liver. Notwithstanding the fact that the mouse is an interesting and essential model to study TMPRSS6 function, mouse isoforms do differ from those found in human. However, we nonetheless focused on characterizing the human isoforms of this protease.

### 3.3 Human TMPRSS6 isoforms have different functionalities

**Figure 4:**
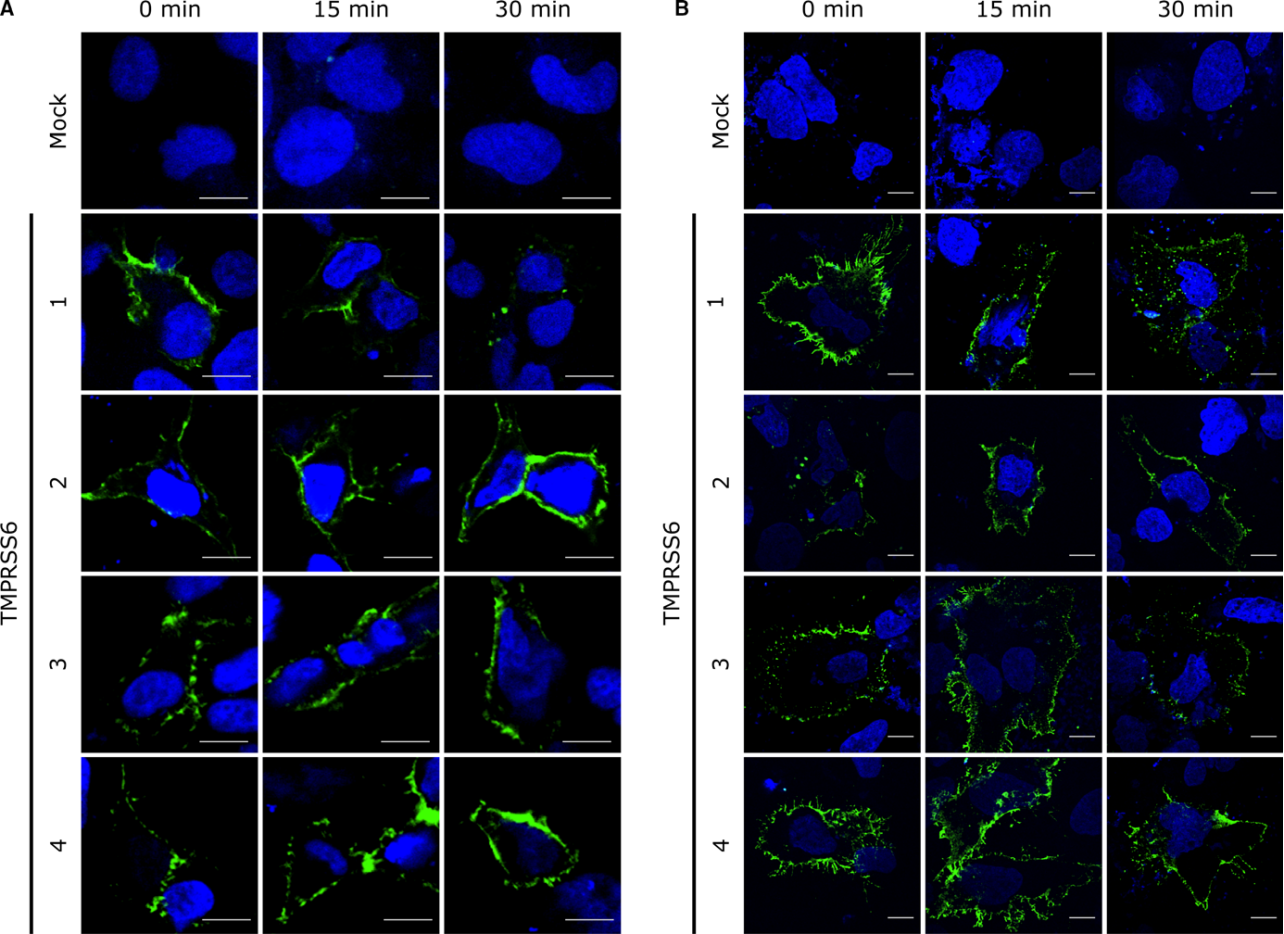
TMPRSS6 isoforms expression at the cell surface and internalization in transfected cells. (A) HEK293 and (B) Hep3B cells were grown on poly‐l‐lysine coverslips and transfected with TMPRSS6‐V5 isoforms. Cells were surface‐labelled with anti‐V5 FITC antibody and incubated for different times (0‐30 min) at 37°C prior to processing for confocal fluorescence microscopy analysis. Anti‐V5 FITC immunofluorescence is displayed in green, and the DAPI stained nucleus is shown in blue (scale bars: 10 μm, n = 3)

We previously showed that TMPRSS6‐1 contained a cell surface internalization motif in its cytoplasmic tail (aa 2‐11),^26^ a motif that is absent in TMPRSS6‐2, TMPRSS6‐3 and TMPRSS6‐4. Thus, we reasoned that TMPRSS6‐2, TMPRSS6‐3 and TMPRSS6‐4 would not be internalized if they were expressed at the cell surface. To assess the ability of TMPRSS6 isoforms to reach the surface and internalize, we used HEK293 transfected cells, a cell line not expressing TMPRSS6 endogenously. This model has already been used by our group to demonstrate TMPRSS6‐1 internalization.^26^ Cells transiently transfected with extracellular, C‐terminal, V5‐tagged TMPRSS6 isoform constructs were surface‐labelled with an anti‐V5‐FITC antibody and kept at 4°C to prevent internalization or incubated at 37°C for 15 or 30 minutes to facilitate constitutive internalization (Figure 4A). Consistent with our previous findings,^26^ TMPRSS6‐1 internalizes and is found in intracellular vesicles after 30 minutes at 37°C. TMPRSS6‐2, TMPRSS6‐3 and TMPRSS6‐4 reach the cell surface but do not internalize. Similar results were also observed in the hepatocellular carcinoma cell line Hep3B, a cell line expressing TMPRSS6 endogenously (Figure 4B).^39^ However, the internalization rate seemed slower in Hep3B cells when compared to HEK293 transfected cells as more TMPRSS6‐1 remain at the cell surface at 30 minutes (Figure 4A, B).

**Figure 5:**
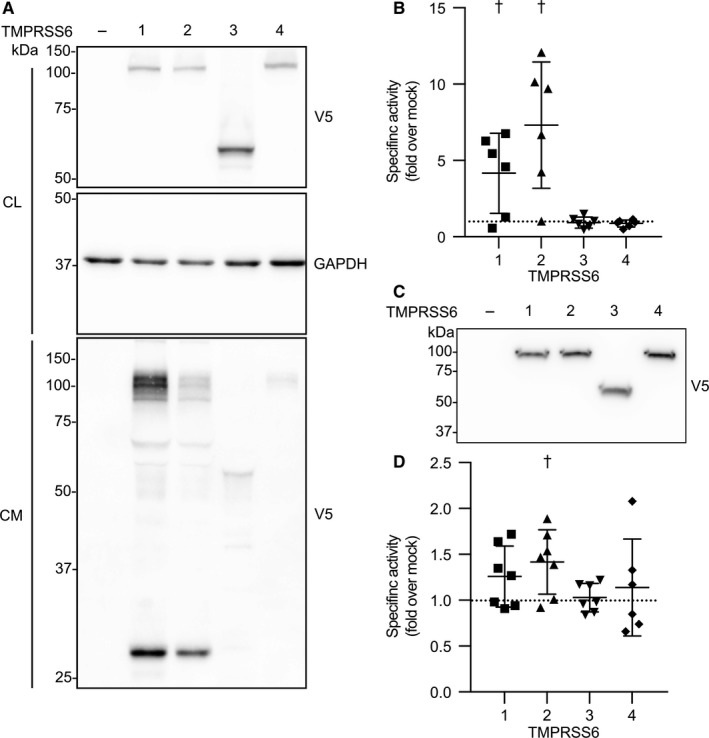
TMPRSS6 isoforms activity. (A) Hep3B cells were transfected with TMPRSS6‐V5 isoforms. Expression was detected by immunoblotting with an anti‐V5 antibody. Equal amounts of cell lysate (CL) and concentrated cell medium (CM) were loaded on 12% SDS‐polyacrylamide gels. Cell lysate GAPDH was blotted as a loading control (n = 3). (B) Proteolytic activity was measured in the cell medium of Hep3B cells transfected with TMPRSS6‐V5 isoforms. The fluorescence released by the cleavage of Boc‐QAR‐AMC (200 μmol/L) was monitored. Results are presented as specific activity (fluorescence units/μL/μg of membrane extracts), are baseline corrected and are shown as scatter plot ± SD (n = 6). (C) Presence of TMPRSS6 isoform in total membranes isolates from Hep3B‐transfected cells was confirmed by immunoblotting TMPRSS6‐V5 isoforms with an anti‐V5 antibody. Equal amounts of membranes were loaded on 12% SDS‐polyacrylamide gels. (D) Proteolytic activity was measured in the membrane fractions of Hep3B cells transfected with TMPRSS6 isoforms. The fluorescence released by the cleavage of Boc‐QAR‐AMC (200 μmol/L) was monitored. Results are presented as specific activity (fluorescence units/μL/μg of membrane extracts), are baseline corrected and are shown as scatter plot ± SD (n ≥ 6)

We next examined the catalytic‐related functionalities of TMPRSS6‐V5‐tagged isoforms in transiently transfected Hep3B cells. Because this cell line is derived from liver (where TMPRSS6 is mostly expressed), it may be a better model to mimic the physiological conditions of the liver. We addressed the ability of TMPRSS6 to be shed from the cell surface, which is an event dependent on TMPRSS6 catalytic activity.^24^, ^26^ Immunoblotting shows that all isoforms are expressed in cell lysates as zymogen forms migrating at >100 kDa for TMPRSS6‐1, TMPRSS6‐2 and TMPRSS6‐4 or at >50 kDa for the truncated TMPRSS6‐3 (Figure 5A, upper panel). Interestingly, and unlike TMPRSS6‐1 and TMPRSS6‐2, TMPRSS6‐4 does not undergo shedding of its catalytic domain (Figure 5A, lower panel). These results confirm those obtained in HEK293‐transfected cells (Figure S4).

The catalytic activities of TMPRSS6 isoforms were then determined in the extracellular medium of Hep3B‐transfected cells (Figure 5B). Proteolytic activity was detected in the media of cells transfected with TMPRSS6‐1 and TMPRSS6‐2 while no activity was observed in the media of cells transfected with TMPRSS6‐3 or TMPRSS6‐4. The same experiment performed in HEK293 cells statistically showed more activity in the cell media of cells transfected with TMPRSS6‐2 than with TMPRSS6‐1 (Figure S4). We have been suggested that the statistically significant differences in activity measured in HEK293 cells media might be caused by the internalization of TMPRSS6‐1, thus allowing more relative proteolytic activity for TMPRSS6‐2 as it does not undergo internalization and remains at the cell surface where it can be shed.

To verify if isoforms can only be proteolytically active at the membrane but not in the media under their shed form, we next isolated total membranes of Hep3B‐transfected cells. Western blot analysis confirms the presence of all TMPRSS6 isoforms in the membranes of transfected cells (Figure 5C). The proteolytic activity in these membrane fractions was then measured (Figure 5D). We detected activity in the membranes fractions of cells transfected with TMPRSS6‐2 while no statistically significant activity was detected for membranes of cells transfected with TMPRSS6‐1, TMPRSS6‐3 and TMPRSS6‐4. This support results obtained in cell media which suggests that isoforms 3 and 4 are inactive or at least, in the case of 4, possesses severely altered activity.

### 3.4 Human TMPRSS6 isoforms interact with HJV

**Figure 6:**
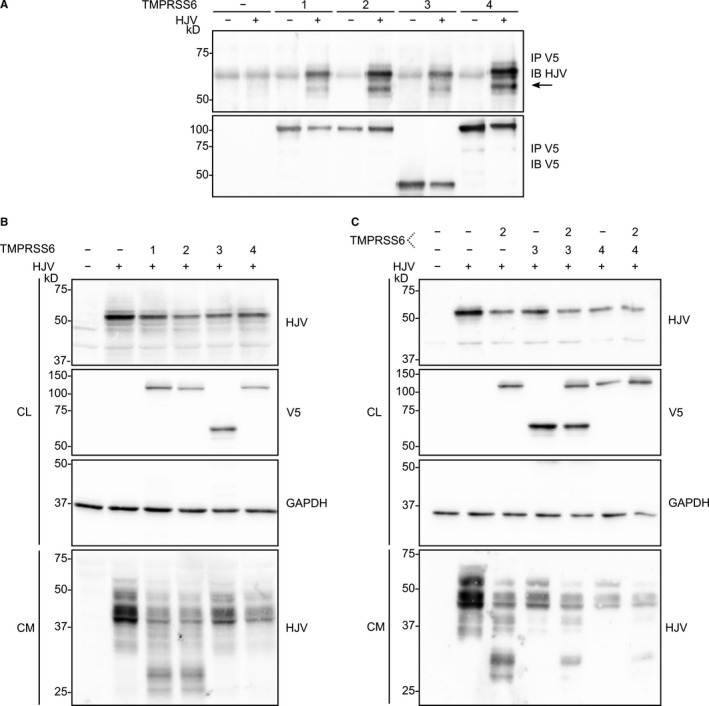
TMPRSS6 isoforms interaction with haemojuvelin. (A) Hep3B cells were cotransfected with TMPRSS6V5‐tagged and haemojuvelin (HJV). Immunoprecipitation was performed in cell lysate using an anti‐V5 antibody. Samples were loaded on 10% SDS‐polyacrylamide gels and immunoblotting was performed using anti‐HJV or anti‐V5 antibodies (n = 3). (B) Hep3B cells were cotransfected with TMPRSS6V5‐tagged isoforms and HJV. HJV cleavage in cell media was detected by immunoblotting with anti‐HJV antibody. Equal amounts of cell lysate (CL) and concentrated cell medium (CM) were loaded on 12% SDS‐polyacrylamide gels. Cell lysate GAPDH was blotted as a loading control (n = 3). (C) Hep3B cells were cotransfected either with HJV alone or in combination with one or two TMPRSS6V5‐tagged isoform. Equal amounts of cell lysate (CL) and concentrated cell medium (CM) were loaded on 12% SDS‐polyacrylamide gels. Cell lysate GAPDH was blotted as a loading control (n = 3)

Even though TMPRSS6‐3 does not have LDLRA or catalytic domains and TMPRSS6‐4 has altered catalytic activity, we examined whether these isoforms interact with HJV.^13^, ^29^ Proteins from Hep3B cells cotransfected with TMPRSS6 isoforms and HJV were immunoprecipitated using an anti‐V5 antibody, and the ability to interact with HJV was verified by immunoblotting with HJV antibodies. Interestingly, all four TMPRSS6 isoforms interacted with HJV as seen by the detected 50 kDa form (Figure 6A). These results indicate that neither TMPRSS6 catalytic domain, as previously suggested,^13^ nor its LDLRAs domains are required for interaction with HJV.

We next assessed the ability of these isoforms to cleave HJV. Immunoblotting of media proteins reveals that when transfected alone, HJV is shed in the media and immunoreactive proteins, ranging from 50 to 30 kDa, can be detected (Figure 6B, lower panel). When cotransfected with TMPRSS6‐1 or TMPRSS6‐2, lower molecular weight bands appear, which indicates proteolytic cleavage.^29^ In contrast, TMPRSS6‐3 and TMPRSS6‐4 do not cleave HJV (Figure 6B, lower panel). Similar results showing TMPRSS6‐HJV interaction and HJV cleavage were also obtained in HEK293‐transfected cells (Figure S4).

Finally, as TMPRSS6‐3 and TMPRSS6‐4 interact with but do not cleave HJV, we assessed their ability to act as dominant negative isoforms. To investigate this hypothesis, Hep3B cells were cotransfected with HJV, proteolytically active TMPRSS6‐2 and TMPRSS6‐3 or TMPRSS6‐4. Immunoblotting of media proteins showed that less proteolytic cleavage of HJV was observed when cells were cotransfected with HJV, TMPRSS6‐2 and TMPRSS6‐3 or TMPRSS6‐4 than only with HJV and TMPRSS6‐2, thus emphasizing the potential properties of TMPRSS6‐3 and TMPRSS6‐4 as dominant negative isoforms able to sequestrate HJV (Figure 6C).

## 4 DISCUSSION

TMPRSS6 is a type II serine protease mainly expressed at the cell surface of hepatocytes and plays an important role in iron regulation, most notably, through HJV cleavage, thus regulating the BMP/SMAD signalling pathway, leading to hepcidin production.^13^, ^14^

Our findings reveal the existence of 4 distinct *TMPRSS6* isoforms and highlight different relative abundance in human tissues. By analysing publicly accessible RNA‐seq data, we demonstrate that *TMPRSS6‐2* has the highest expression in human liver and should be considered the main liver isoform. We also show that *TMPRSS6‐1*, which is considered the canonical variant according to UniProt Consortium,^21^ is expressed in testis at low levels and not detected in the liver using RNA‐seq and RT‐PCR. Interestingly, using a combination of techniques, we show that *TMPRSS6‐3* (lacking LDLRA and catalytic domains) is expressed at very low levels in liver but is expressed in testis. Importantly, we report that *TMPRSS6‐4* is expressed in human liver which suggests that this isoform could have a significant role in hepatic functions.

We also reveal the existence of three *Tmprss6* mouse isoforms and studied their expression performed with RNA‐seq data analysis of mouse livers. We also confirmed the expression of these isoforms using PCR amplification on mice primary hepatocytes. The differences between mice *Tmprss6* isoforms are subtle but will ultimately need to be investigated. Moreover, it is important to note that at the present time, there is less data available for mice than for humans and therefore mice annotations may not be as reliable as human annotations in databases. Thus, the possibility that other *Tmprss6* isoforms exist cannot be ruled out.

Using heterologous expression in HEK293 and Hep3B cells, we show that all human TMPRSS6 isoforms reach the cell surface. While TMPRSS6‐1 undergoes constitutive internalization, as previously described by our group,^26^ TMPRSS6‐2, TMPRSS6‐3 and TMPRSS6‐4 remain at the plasma membrane. We also describe the inability of TMPRSS6‐4 to undergo auto‐activation, which leads to the production of a catalytically altered protein unable to cleave HJV in the cell media compared to TMPRSS6‐1 and TMPRSS6‐2. Importantly and regardless of the presence of catalytic domains or activity, we show that TMPRSS6‐3 and TMPRSS6‐4 interact with HJV. This finding correlates with the results of Silvestri et al.^13^ and suggests that TMPRSS6 ectodomains are involved in interactions with HJV. Moreover, we show that TMPRSS6‐3 and TMPRSS6‐4 act as dominant negative regulators of HJV cleavage by TMPRSS6. We believe that, similar to the TMPRSS6 mask mutant,^13^ TMPRSS6‐3 and TMPRSS6‐4 could represent a novel mechanism of iron regulation. Indeed, similar to TMPRSS6‐3, the catalytically truncated TMPRSS6 mask mutant interacts with HJV^13^ but does not repress hepcidin promoter activation.^40^ Moreover, as TMPRSS6 proteolytic activity has been previously described as protective towards prostate cancer in vitro,^41^ truncated *TMPRSS6‐3* or functionally altered *TMPRSS6‐4* expression could be related to cancer development and their role in cancers should be thoroughly studied.

Therefore, transcriptional regulation of human isoforms under different conditions could be of great interest in both iron regulation and cancer‐related contexts. The effect of factors known to regulate *TMPRSS6* expression, such as IL‐6, LPS and BMP‐6,^39^, ^42^ should be taken into consideration in human hepatoma cell lines, but also in mice to see if they affect expression levels of specific *TMPRSS6* isoforms.

Of note, we had previously demonstrated^26^ (and validated herein) that only TMPRSS6‐1 undergoes internalization when expressed in two heterologous model, one of which being primary hepatocytes. Therefore, we believed isoform 1 was expressed in the liver. Because our present results reveal that isoform 1 is not detected in liver, we believe our previous data,^26^ which used high concentrations of IgG antibodies (800 nmol/L) to label cell surface TMPRSS6, might therefore be a consequence of internalization by the IgG receptor FcRn (FCGRT), known to be expressed in both hepatocytes^43^ and HepG2 cells.^33^ On the other hand, it is clear that residues 1‐9 encoded by exon 1 are responsible for the internalization of that isoform as validated when the isoform is expressed using heterologous expression in HEK293 and Hep3B cell lines.

Taken together, our results highlight the importance of identifying which *TMPRSS6* isoforms are expressed in human tissues as well as the properties these isoforms possess. Considering the important role of TMPRSS6 in iron regulation, the protease isoforms herein described and studied should be taken into account in future studies, especially for TMPRSS6‐3 and TMPRSS6‐4, for which physiological functions are still to be elucidated.

## CONFLICT OF INTEREST

The authors declare no competing financial interests.

## AUTHORS CONTRIBUTION

S.P.D. and F.B. contributed equally to this work. S.P.D., F.B., A.D. and R.L. designed the study. F.B. performed the RNA‐sequencing data analysis and S.P.D. conducted the biological characterization of isoforms. A.D. performed the membrane isolations. S.P.D. and M.G.G. performed the mice primary hepatocyte isolation. S.P.D., F.B., A.D. and R.L. wrote the manuscript.
