# Supplementary material for: Transcriptome analysis reveals TMPRSS6 isoforms with distinct functionalities
Source: J Cell Mol Med. 2018 Feb 14;22(4):2498–509. doi: 10.1111/jcmm.13562 (PMC5867103; doi:10.1111/jcmm.13562)
Supplement: Supplementary file 1 [file JCMM-22-2498-s001.docx]

**Supplementary information**

**Materials and methods**

**Cells, Antibodies, and Reagents**

HEK293 and Hep3B cells were purchased from American Type Culture Collection (Manassas, VA). HEK293 and Hep3B cells were respectively maintained in high glucose Dulbecco’s Modified Eagle’s Medium (DMEM) or Eagle’s Minimum Essential Medium (EMEM) both containing 10% fetal bovine serum, 2 mM L-glutamine, 100 IU/ml penicillin and 100 µg/ml streptomycin or serum-free media HCELL-100 (WISENT, St-Bruno, Canada). Poly-L-lysine coated coverslips were from Corning (Bedford, MA). Restriction enzymes XhoI and KpnI-HF were from New England Biolabs (Ipswich, MA). Anti-V5, HRP and FITC-linked Anti-V5 monoclonal antibodies (mAb) were from Invitrogen (Waltham, MA). HRP-linked Anti-GAPDH rabbit mAb was from Cell Signaling Technology (Danvers, MA). Goat polyclonal anti-Hemojuvelin antibody and t-butoxycarbonyl-Gln-Ala-Arg-7-amino-4-methylcoumarin (Boc-QAR-AMC) were purchased from R&D Systems (Minneapolis, MN). Lipofectamine 3000 was from Invitrogen (Carlsbad, CA). Centrifugal filters were from Merck Millipore (Cork, Ireland). Lysis buffer (1% Triton, 50 mM Tris, 150 mM NaCl, 5 mM EDTA) was supplemented with protease inhibitor from Roche (Mannhem, Germany). Protein A/G PLUS-agarose beads were from Santa-Cruz Biotechnology (Dallas, TX). C57BL/6 WT mice were from Charles River (Montréal, Canada). Ketamine was from Vétoquinol (Lavaltrie, Canada). Xylazine was from Bimeda (Cambridge, Canada). Surflo Winged Infusion Set was from Terumo (Tokyo, Japan). Liver Perfusion Medium, Liver Digest Medium, Hepatocyte Wash Medium and William’s E Medium (supplemented with Primary Hepatocyte Thawing and Plating Supplements) were from Life Technologies (Grand Island, NY). Percoll was from GE Healthcare (Uppsala, Sweden). TRIzol was from Life Technologies (Carlsbad, CA). Liver cDNA pool was from (BioChain, Newark CA). ProteoExtract Native Membrane Extraction Kit was from Millipore (Darmstadt, Germany).

**Mice primary hepatocytes**

Mice primary hepatocytes were obtained from C57BL/6 mice. Mice were anesthetized with ketamine/xylazine (87/13 mg) intraperitoneally before the abdomen was opened. The inferior vena cava was blocked between the heart and the liver and liver portal vein connected to the perfusion system using a Surflo Winged Infusion Set. The inferior vena cava was then cut in the lower abdomen to allow the flow to exit the liver. The liver was perfused with 70 mL of Liver Perfusion Medium with an initial flow of 4.5 mL/min raised to 8 mL/min after 1 min. The liver was then perfused with Liver Digest Medium at 8 mL/min. Liver was then cut into small pieces and dissociated in 20 mL of Hepatocyte Wash Medium (HWM). This suspension was then passed on a 70 µm filter and washed with 20 mL of HWM. Cells were centrifuged at 50g for 3 min at 4˚C. The pellet was suspended in 40% Percoll (diluted in HWM). Cells were centrifuged at 200g for 7 min at 4˚C. The pellet, composed of the viable hepatocytes, was then washed twice with 20 mL of HWM, suspended in 10 mL of plating medium and cells were counted using an hemacytometer. Cells were plated in 6 wells plate (5x10^5^ cells/well). The next day cells were washed with PBS. Cells were treated with Trizol reagent to further isolate and analyze RNA. The use of animals in the context of this project was approved by the Université de Sherbrooke Animal Ethic Committee.

**Plasmid constructions and Site-directed Mutagenesis**

TMPRSS6-1 and HJV cDNA were obtained and cloned as previously described [1]. TMPRSS6-2 and construct was obtained using QuikChange Lightning Site-Directed Mutagenesis Kit as recommended by the manufacturer (Agilent Technologies, Santa Clara, CA). Primers used are listed in Table S6. TMPRSS6-3 construct was obtained by insertion of a gBlock fragment (Integrated DNA Technologies, Coralville, IA) using NEBuilder HiFi DNA Assembly Cloning Kit (New England Biolabs) as described by the manufacturer in a linearized (XhoI and KpnI cleavage) modified form of pcDNA6/V5-His (Invitrogen) previously described[1]. Nucleotides coding for residues 1-9 were then removed using QuikChange Lightning Site-Directed Mutagenesis Kit as recommended by the manufacturer. TMPRSS6-4 construct was obtained by the insertion of a gBlock fragment using QuikChange II XL Site-Directed Mutagenesis Kit (Agilent Technologies). gBlock fragments are listed in Table S7.

**Immunofluorescence**

HEK293 and Hep3B cells were seeded on 12 mm poly-L-lysine coated coverslips in 6-well plates. Cells were transfected with TMPRSS6 isoform plasmid DNA using Lipofectamine 3000. 24h post-transfection, cells were surface labelled with anti-V5-FITC antibody for 1h at 4˚C in DMEM 0.5% FBS. Cells were washed and incubated at 37˚C in DMEM 10% FBS for 15 or 30 min. Cells were then prepared as previously described [2] and mounted using ProLong Diamond Antifade Mountant with DAPI (Invitrogen, Eugene, OR). Cells were examined using a Plan Apo 60x oil immersion objective NA 1.42 on inverted spectral scanning confocal microscope FV1000 (Olympus, Tokyo, Japan). Cells were laser-excited at 405 nm (50 mW Violet diode laser) and 488 nm (40 mW Blue Argon Laser). Images were pseudocolored according to their original fluorochrome and merged using FluoView software (Olympus, Tokyo, Japan).

**Expression and detection of TMPRSS6**

HEK293 or Hep3B cells were transfected TMPRSS6 isoform DNA using Lipofectamine 3000 in 6-well plates. After 24h transfection, cell media was replaced with HCELL-100 media for 24h. Cell media were collected and cells were lysed. 1 mL of cell media was concentrated using centrifugal filters (21,100 *g*, 15 min, 4˚C). Samples were loaded on 12% SDS-polyacrylamide gels and analyzed by immunoblotting with anti-V5 antibody.

**Proteolytic activity measurements**

HEK293 or Hep3B cells were transfected respectively with TMPRSS6 isoform DNA. After a 24h transfection, cell media was replaced with HCELL-100 media for another 24h. Media were collected and proteolytic activity was measured by the release of fluorescence (excitation, 360 nm; emission 460 nm) with 200 µM Boc-QAR-AMC for 1h at room temperature in an FLx800 TBE microplate reader (Bio-Tek Instruments, Winooski, VT). HEK293 proteolytic activities were corrected for the total lysate proteins while Hep3B proteolytic activities were corrected for membrane isolated proteins.

**Hemojuvelin processing by TMPRSS6**

HEK293 or Hep3B cells were co-transfected in 6-well plates with one or two TMPRSS6 isoform DNA and hemojuvelin transcript A using Lipofectamine 3000. After a 24h transfection, cell media was replaced with HCELL-100 media for another 24h. Cell media were collected and cells lysed. Cell media were concentrated using centrifugal filters. Samples were loaded on 12% SDS-polyacrylamide gels and analyzed by immunoblotting with anti-hemojuvelin antibody.

**Interaction between TMPRSS6 and HJV**

In 6-well plates, HEK293 or Hep3B cells were co-transfected with TMPRSS6 isoform DNA and hemojuvelin transcript A using Lipofectamine 3000. After a 24h transfection, cells were washed and harvested on ice in 300 µL lysis buffer for 15 min. Protein samples were immunoprecipitated with anti-V5 antibody and Protein A/G PLUS-agarose beads for 24h at 4˚C. Samples were then transferred onto Pierce Spin Column (Pierce Biotechnology, Rockford, IL) and centrifuged (5 min, 6,200 *g*, 4˚C) and washed. Immunoprecipitated proteins were eluted in 30 µL Laemmli buffer (0.4% 2-Mercaptoethanol), loaded on 12% SDS-polyacrylamide gels and analyzed by immunoblotting hemojuvelin.

**Supplemental references**

[1] **Béliveau F, Brulé C, Désilets A, et al.** Essential role of endocytosis of the type II transmembrane serine protease TMPRSS6 in regulating its functionality. *J. Biol. Chem.* 2011; 286; 29035–43.

[2] **Brodeur J, Larkin H, Boucher R, et al.** Calnuc binds to LRP9 and affects its endosomal sorting. *Traffic* 2009; 10; 1098–114.

**Supplemental tables**

**Table S1:** **Datasets used from the Genotype-Tissue Expression (GTEx) Project.**

| **GTEx samples id** | | |
| --- | --- | --- |
| **Liver** | **Pituitary** | **Testis** |
| GTEX-T6MN-1226-SM-3NMA5 | GTEX-WVLH-3126-SM-3MJGA | GTEX-OOBK-2126-SM-3LK5T |
| GTEX-11NV4-1326-SM-5HL6V | GTEX-11NUK-3126-SM-5986M | GTEX-11ZUS-2726-SM-5FQUA |
| GTEX-131XH-0626-SM-5LZWH | GTEX-13OW7-3126-SM-5L3HB | GTEX-QEG4-0126-SM-48TZE |
| GTEX-RN64-1826-SM-48FDV | GTEX-13OVL-3226-SM-5L3H8 | GTEX-11TUW-2226-SM-5EQL9 |
| GTEX-13VXU-0926-SM-5IFFH | GTEX-139TS-2926-SM-5KM1Z | GTEX-UPJH-0126-SM-4IHLL |
| GTEX-11NUK-1226-SM-5P9GM | GTEX-11DYG-3126-SM-5A5L6 | GTEX-N7MS-0126-SM-3TW8O |
| GTEX-13FTZ-0726-SM-5IFFY | GTEX-PVOW-2726-SM-48TCA | GTEX-T6MN-2026-SM-4DM7L |
| GTEX-13SLX-1226-SM-5S2Q6 | GTEX-1192W-3026-SM-5GZZV | GTEX-Z93S-1726-SM-5HL8G |
| GTEX-ZYT6-0626-SM-5E45V | GTEX-1212Z-2926-SM-59HKV | GTEX-11LCK-2326-SM-5HL53 |
| GTEX-WZTO-0626-SM-4PQYY | GTEX-N7MS-2625-SM-3LK77 | GTEX-XAJ8-1326-SM-47JYT |
| GTEX-QEG4-1826-SM-4R1JN | GTEX-12WSB-3226-SM-59HJP | GTEX-QV31-1126-SM-4R1K4 |
| GTEX-13NYB-1026-SM-5IFH3 | GTEX-13OVH-3126-SM-5MR4Z | GTEX-ZQUD-2026-SM-51MSM |
| GTEX-ZVT3-1626-SM-5GU66 | GTEX-WL46-3026-SM-3LK7Z | GTEX-WZTO-0326-SM-4PQYZ |
| GTEX-11DXY-0526-SM-5EGGQ | GTEX-R55E-2726-SM-48FCX | GTEX-111VG-1926-SM-5GIDO |
| GTEX-Q2AG-1126-SM-48U1P | GTEX-11NV4-2326-SM-5BC4X | GTEX-WK11-0326-SM-4OOS6 |
| GTEX-QESD-2026-SM-447BI | GTEX-T6MN-2726-SM-4DM77 | GTEX-OOBJ-2126-SM-3NB1N |
| GTEX-XBEC-1526-SM-4AT68 | GTEX-11ONC-2826-SM-5P9GQ | GTEX-QEG5-0126-SM-4R1JR |
| GTEX-SJXC-1226-SM-4DM78 | GTEX-1399T-2926-SM-5IFF8 | GTEX-139TS-1726-SM-5IJG5 |
| GTEX-11ZUS-2526-SM-59872 | GTEX-145MH-3126-SM-5S2QT | GTEX-11NUK-2626-SM-5A5MB |
| GTEX-131YS-1626-SM-5HL6C | GTEX-13OW8-3026-SM-5L3G8 | GTEX-XQ3S-2726-SM-4BOP2 |
| GTEX-145MF-0826-SM-5QGQA | GTEX-13OW6-3126-SM-5L3IA | GTEX-11OF3-1826-SM-5987N |
| GTEX-YECK-1926-SM-4W21H | GTEX-13FHO-3126-SM-5IJF6 | GTEX-WOFM-1126-SM-4OOSB |
| GTEX-145LU-1326-SM-5LU9N | GTEX-14BMV-3126-SM-5S2UV | GTEX-111FC-1926-SM-5GZYC |
| GTEX-1399T-0826-SM-5IFES | GTEX-1313W-3226-SM-5LZUU | GTEX-11NV4-1726-SM-5N9FC |
| GTEX-12WSI-0226-SM-5GCNA | GTEX-XLM4-3126-SM-4AT6M | GTEX-11WQC-2326-SM-5EQKE |
| GTEX-11OF3-0726-SM-5BC4Z | GTEX-131XW-3226-SM-5LZUO | GTEX-REY6-0126-SM-48FDT |
| GTEX-13112-1426-SM-5EGH8 | GTEX-139TT-2726-SM-5IJFM | GTEX-YEC3-1726-SM-5IFIK |
| GTEX-PX3G-0826-SM-48TZS | GTEX-145MO-3126-SM-5S2RT | GTEX-X5EB-2026-SM-4E3KA |
| GTEX-RNOR-1426-SM-48FDJ | GTEX-11ZUS-3026-SM-5EGI4 | GTEX-12WSH-0326-SM-5GCNH |
| GTEX-RTLS-1326-SM-46MUN | GTEX-12ZZY-3126-SM-5DUX3 | GTEX-U8XE-0126-SM-4E3I3 |
| GTEX-13FLV-0326-SM-5N9DJ | GTEX-117YW-2826-SM-5H116 | GTEX-S95S-1126-SM-4B64E |
| GTEX-1212Z-0226-SM-59HLF | GTEX-13VXU-3126-SM-5SIA4 | GTEX-11GS4-2026-SM-5N9CP |
| GTEX-12WSM-0726-SM-5GCOW | GTEX-WHSE-3126-SM-3P5ZI | GTEX-PW2O-1426-SM-48TCD |
| GTEX-11WQC-0726-SM-5EQMR | GTEX-13G51-3226-SM-5IFG7 | GTEX-111YS-2026-SM-5EGGL |
| GTEX-11ZTS-1426-SM-5EQMM | GTEX-11GS4-3026-SM-5A5LG | GTEX-117YW-1526-SM-5EGGP |
| GTEX-Y5V5-0926-SM-4VBPZ | GTEX-145MF-2626-SM-5O98S | GTEX-11O72-0726-SM-5P9GO |
| GTEX-14DAQ-1726-SM-5S2R2 | GTEX-ZAK1-3126-SM-5S2O8 | GTEX-Y111-2426-SM-4TT23 |
| GTEX-1399R-1226-SM-5P9GF | GTEX-13CF2-3226-SM-5IFF6 | GTEX-13N2G-0126-SM-5N9DV |
| GTEX-13QJC-0726-SM-5RQJK | GTEX-13FTY-2826-SM-5J2ML | GTEX-OHPM-2126-SM-3LK75 |
| GTEX-139TU-0826-SM-5IJFG | GTEX-13N2G-2926-SM-5IJEE | GTEX-14ABY-0626-SM-5Q5C9 |
| GTEX-12WSD-1426-SM-5GCN9 | GTEX-12WSC-3126-SM-5GCNB | GTEX-13O1R-0726-SM-5IJEI |
| GTEX-14AS3-0126-SM-5Q5F4 | GTEX-WZTO-3026-SM-3NMA2 | GTEX-S7PM-0626-SM-4AD4Q |
| GTEX-147JS-1126-SM-5RQIW | GTEX-14753-2926-SM-5LU9J | GTEX-14753-0626-SM-5Q5CY |
| GTEX-11DXZ-0126-SM-5EGGY | GTEX-11EMC-3126-SM-5EGJP | GTEX-144GL-0726-SM-5LU4P |
| GTEX-139TS-1426-SM-5IFJD | GTEX-11DXY-3026-SM-5N9CB | GTEX-U8T8-1126-SM-4DXUE |
| GTEX-UPIC-0926-SM-4IHLV | GTEX-131YS-3226-SM-5IFGV | GTEX-13112-0226-SM-5P9IV |
| GTEX-13OW6-2626-SM-5IFF2 | GTEX-13N1W-3126-SM-5IFHB | GTEX-X3Y1-2626-SM-4PQZI |
| GTEX-147F4-1426-SM-5LUA8 | GTEX-13JUV-2626-SM-5N9EB | GTEX-SNOS-1126-SM-4DM67 |
| GTEX-12ZZZ-1326-SM-59HKW | GTEX-12126-1126-SM-5P9GP | GTEX-13OW5-2526-SM-5L3I1 |
| GTEX-RWSA-1426-SM-47JXA | GTEX-147GR-2926-SM-5RQIN | GTEX-12ZZY-0126-SM-5LZV2 |
| GTEX-11GSP-0626-SM-5986T | GTEX-148VJ-2926-SM-5Q5DA | GTEX-12C56-1426-SM-5FQSW |
| GTEX-14753-1626-SM-5NQ9L | GTEX-OXRN-2626-SM-48TBX | GTEX-ZVZP-2226-SM-57WBF |
| GTEX-X4EO-1126-SM-4QARQ | GTEX-13112-3126-SM-5IFGP | GTEX-P4QS-2126-SM-3NMCF |
| GTEX-X4EP-1026-SM-4QAS5 | GTEX-13RTJ-2926-SM-5Q5DE | GTEX-TKQ1-0926-SM-4DXU2 |
| GTEX-XOTO-0826-SM-4B65O | GTEX-11ZTS-3326-SM-5LU9Y | GTEX-ZYT6-2726-SM-5GICP |
| GTEX-ZF2S-3026-SM-4WWCH | GTEX-13FHP-3126-SM-5KLYX | GTEX-12WSL-2326-SM-5DUXQ |
| GTEX-12KS4-1326-SM-5LUB3 | GTEX-1477Z-2826-SM-5SI9J | GTEX-11EQ9-1926-SM-5PNVV |
| GTEX-X3Y1-2726-SM-4PQZH | GTEX-11DXW-1226-SM-5H133 | GTEX-11NSD-1026-SM-5N9BE |
| GTEX-11ZVC-0726-SM-5FQT9 | GTEX-14BIL-2926-SM-5SIAF | GTEX-U3ZM-1626-SM-4DXSK |
| GTEX-13NZB-0626-SM-5IFH6 | GTEX-113JC-2826-SM-5EGIT | GTEX-XBEC-0126-SM-4GIDT |
| GTEX-TKQ2-1726-SM-4DXUP | GTEX-12WSD-3226-SM-5HL9G | GTEX-13NZA-2526-SM-5IJFX |
| GTEX-14A5I-1726-SM-5QGQ5 | GTEX-T5JC-2526-SM-4DM6G | GTEX-T5JC-0726-SM-4DM55 |
| GTEX-R53T-0326-SM-48FEC | GTEX-12ZZW-3026-SM-5LZW7 | GTEX-ZVTK-0126-SM-57WDG |
| GTEX-132NY-0926-SM-5P9G3 | GTEX-139T4-0826-SM-5N9GF | GTEX-OXRL-2126-SM-3NM98 |
| GTEX-RU72-1426-SM-46MUF | GTEX-14A5H-3026-SM-5RQHV | GTEX-ZAB5-2426-SM-5CVMW |
| GTEX-13O3O-1826-SM-5IFGW | GTEX-13JVG-3026-SM-5IJEV | GTEX-Y5V6-1726-SM-4VDSZ |
| GTEX-ZAB5-0426-SM-5CVMI | GTEX-13SLX-3026-SM-5RQJU | GTEX-TKQ2-1526-SM-4DXUN |
| GTEX-ZVP2-0626-SM-51MSO | GTEX-117XS-2926-SM-5N9BX | GTEX-Q2AH-1526-SM-48TZG |
| GTEX-ZVT4-0626-SM-5E45T | GTEX-RNOR-2426-SM-48FDY | GTEX-XPVG-2226-SM-4B65U |
| GTEX-QV44-0326-SM-4R1KD | GTEX-T2IS-3126-SM-32QPK | GTEX-13VXU-0726-SM-5J2O7 |
| GTEX-13N2G-0926-SM-5IFGJ | GTEX-12584-3126-SM-5EGKR | GTEX-Q2AI-1226-SM-48U14 |
| GTEX-X261-1726-SM-4PQYT | GTEX-13NYS-2926-SM-5IFGH | GTEX-S7SE-0326-SM-4AT5Q |
| GTEX-YB5E-0326-SM-5IFHU | GTEX-13OW5-2926-SM-5IJF2 | GTEX-T8EM-0126-SM-4DM5R |
| GTEX-11EQ9-0526-SM-5A5JZ | GTEX-11ZVC-3226-SM-5FQV1 | GTEX-ZYFC-0126-SM-5GIEH |
| GTEX-WFON-1726-SM-4LVMQ | GTEX-13SLW-2926-SM-5Q5BQ | GTEX-V1D1-2126-SM-4JBH4 |
| GTEX-ZZPU-0426-SM-5GZYH | GTEX-11TUW-3026-SM-5EGKM | GTEX-V955-1926-SM-4KL1L |
| GTEX-YEC4-0826-SM-5P9FV | GTEX-NPJ7-2926-SM-3MJGQ | GTEX-11P82-1526-SM-5BC5M |
| GTEX-REY6-1226-SM-48FDR | GTEX-13PLJ-1726-SM-5L3FT | GTEX-RWSA-2426-SM-47JXR |
| GTEX-WYVS-1926-SM-4PQZ2 | GTEX-11OF3-3026-SM-5GU52 | GTEX-13N1W-2626-SM-5IJEP |
| GTEX-ZF29-2026-SM-4WWB7 | GTEX-RU72-3126-SM-46MUB | GTEX-WH7G-1926-SM-4LVMM |
| GTEX-OOBJ-0826-SM-3NB2K | GTEX-UTHO-3126-SM-3P5ZB | GTEX-117XS-2026-SM-5GID1 |
| GTEX-Q734-0326-SM-48U15 | GTEX-13O1R-3226-SM-5KM58 | GTEX-ZZ64-1126-SM-5GZXY |
| GTEX-13FTW-1126-SM-5J2NV | GTEX-ZYY3-3226-SM-5SI9X | GTEX-11DXZ-2126-SM-59881 |
| GTEX-12696-0826-SM-5EGGE | GTEX-ZAB4-3126-SM-57WCJ | GTEX-13FHP-2826-SM-5IJFW |
| GTEX-P78B-1326-SM-3P611 | GTEX-Q2AG-3026-SM-48U1L | GTEX-12696-0226-SM-5EGL3 |
| GTEX-ZAB4-0826-SM-5LU9D | GTEX-Y111-2926-SM-4TT25 | GTEX-13OVH-0726-SM-5N9BU |
| GTEX-12WSG-0626-SM-5FQTQ | GTEX-13X6K-3026-SM-5QGPA | GTEX-11EQ8-1426-SM-5EGJR |
| GTEX-RM2N-1926-SM-48FCU | GTEX-12696-3126-SM-5FQUX | GTEX-ZDYS-1326-SM-5IJFF |
| GTEX-13113-1326-SM-5GCOI | GTEX-N7MT-1026-SM-3TW8T | GTEX-13FLW-2126-SM-5N9FD |
| GTEX-1497J-0726-SM-5Q5D1 | GTEX-13OVJ-2626-SM-5IJFT | GTEX-XPT6-1626-SM-4B655 |
| GTEX-14E1K-0326-SM-5S2PE | GTEX-TSE9-3126-SM-4DXSY | GTEX-131XE-0426-SM-5IJF4 |
| GTEX-UPK5-1426-SM-4JBHH | GTEX-X4EP-3226-SM-3P5YR | GTEX-R55D-0126-SM-48FEL |
| GTEX-1269C-0626-SM-5FQSS | GTEX-ZAJG-3226-SM-5HL9V | GTEX-XMK1-2026-SM-4B65K |
| GTEX-YFC4-1526-SM-5IFJS | GTEX-11O72-3026-SM-5986R | GTEX-ZUA1-2726-SM-59HLJ |
| GTEX-144GM-1326-SM-5LU5E | GTEX-YFC4-3226-SM-5CVM2 | GTEX-144GM-0426-SM-5Q5C8 |
| GTEX-UTHO-2426-SM-4JBHD | GTEX-X261-3226-SM-3NMC3 | GTEX-ZTSS-1526-SM-51MTC |
| GTEX-145MO-2326-SM-5NQ9K | GTEX-11WQK-3226-SM-5EGI3 | GTEX-R55E-0726-SM-48FCZ |
| GTEX-13PVR-0126-SM-5S2PY | GTEX-14BIM-3226-SM-5RQIQ | GTEX-1212Z-0326-SM-5FQSJ |
| GTEX-14C38-1526-SM-5RQJ7 | GTEX-X585-3126-SM-4QASH | GTEX-WVLH-2626-SM-4MVNV |
| GTEX-VUSG-0126-SM-4KL1X | GTEX-1192X-3026-SM-5N9BM | GTEX-132QS-1226-SM-5P9GD |
| GTEX-S33H-1626-SM-4AD68 | GTEX-Z93S-3026-SM-5CVMS | GTEX-QMRM-1526-SM-4R1K6 |
| GTEX-S4Z8-0526-SM-4AD4T | GTEX-NPJ8-1426-SM-3MJHR | GTEX-ZA64-1626-SM-5CVME |
| GTEX-QEL4-1226-SM-447A4 | GTEX-12WSM-2226-SM-5DUVS | GTEX-13111-1526-SM-5EGJX |
| GTEX-139YR-0226-SM-5IFEM |  | GTEX-X261-2326-SM-4PQYU |
| GTEX-WQUQ-1926-SM-4OOSA |  | GTEX-12WSI-2126-SM-5GCMV |
| GTEX-ZPU1-0826-SM-57WG2 |  | GTEX-11EI6-2226-SM-5EGJM |
| GTEX-X4XY-1626-SM-46MVN |  | GTEX-13O21-1226-SM-5J2MK |
| GTEX-13N11-0926-SM-5IJG2 |  | GTEX-SNMC-1026-SM-4DM7K |
| GTEX-ZYY3-0626-SM-5NQ6W |  | GTEX-S3XE-1526-SM-4AD5A |
| GTEX-146FH-1526-SM-5NQBU |  | GTEX-13NZB-2026-SM-5MR4M |
| GTEX-ZTPG-1426-SM-51MT3 |  | GTEX-U4B1-1526-SM-4DXSL |
| GTEX-Y5LM-0426-SM-4VBRO |  | GTEX-ZT9W-2226-SM-57WFU |
| GTEX-Z9EW-0426-SM-5CVM9 |  | GTEX-RUSQ-2126-SM-47JXK |
| GTEX-U3ZN-0226-SM-3DB8D |  | GTEX-13OVL-0426-SM-5IFG6 |
| GTEX-12WSL-0226-SM-5CVMJ |  | GTEX-QLQW-1026-SM-447A9 |
| GTEX-13NZ9-1326-SM-5MR3V |  | GTEX-SUCS-1326-SM-4DM5T |
| GTEX-131XE-0326-SM-5LZVO |  | GTEX-XBED-2026-SM-4AT5D |
| GTEX-S32W-1926-SM-4AD63 |  | GTEX-ZV7C-2026-SM-5NQ8F |
| GTEX-QDVN-0826-SM-48TZ2 |  | GTEX-YB5E-1926-SM-5IFIG |
|  |  | GTEX-13O61-2026-SM-5J2M6 |
|  |  | GTEX-XGQ4-2026-SM-4AT6G |
|  |  | GTEX-1339X-1926-SM-5PNVP |
|  |  | GTEX-145MO-0126-SM-5S2QU |
|  |  | GTEX-YEC4-1526-SM-4W1YU |
|  |  | GTEX-R55C-1426-SM-48FED |
|  |  | GTEX-111CU-1726-SM-5EGHM |
|  |  | GTEX-XLM4-1526-SM-4AT6D |
|  |  | GTEX-147JS-0126-SM-5S2TW |
|  |  | GTEX-139T6-1226-SM-5IFFC |
|  |  | GTEX-144GN-1626-SM-5Q5BU |
|  |  | GTEX-11WQK-2826-SM-5EQKH |
|  |  | GTEX-QLQ7-1426-SM-4R1JX |
|  |  | GTEX-S4Q7-1226-SM-4AD5I |
|  |  | GTEX-RN64-2326-SM-48FDW |
|  |  | GTEX-ZT9X-1426-SM-5DUX1 |
|  |  | GTEX-11P7K-1026-SM-5HL6Y |
|  |  | GTEX-WFG8-1926-SM-4LVM1 |
|  |  | GTEX-WHSB-2126-SM-4M1XF |
|  |  | GTEX-145MH-2326-SM-5O9AW |
|  |  | GTEX-ZPU1-2126-SM-57WED |
|  |  | GTEX-NFK9-0126-SM-3LK5H |
|  |  | GTEX-13FTW-1326-SM-5LZZD |
|  |  | GTEX-11ONC-2226-SM-5HL6D |
|  |  | GTEX-U3ZH-1526-SM-4DXV1 |
|  |  | GTEX-11TT1-2226-SM-5GU6B |
|  |  | GTEX-YF7O-2026-SM-4W1YE |
|  |  | GTEX-OIZH-2126-SM-3NB1P |
|  |  | GTEX-145LT-0426-SM-5LUAP |
|  |  | GTEX-YJ89-0626-SM-4TT3Z |
|  |  | GTEX-147F4-0626-SM-5LUAK |
|  |  | GTEX-13NYB-2226-SM-5MR58 |
|  |  | GTEX-139TT-2226-SM-5LZWO |
|  |  | GTEX-S33H-0126-SM-4AD62 |
|  |  | GTEX-Y8E4-2226-SM-5LU94 |
|  |  | GTEX-WFON-2026-SM-4LVMW |
|  |  | GTEX-RM2N-1326-SM-48FCW |
|  |  | GTEX-1399R-1626-SM-5P9GG |
|  |  | GTEX-VJYA-1426-SM-4KL1Y |
|  |  | GTEX-ZTX8-1126-SM-51MRM |
|  |  | GTEX-QV44-1726-SM-4R1KG |
|  |  | GTEX-ZAB4-0126-SM-5CVMG |
|  |  | GTEX-Y9LG-1726-SM-4VBQE |
|  |  | GTEX-12WSM-1326-SM-5GCP9 |
|  |  | GTEX-O5YT-2126-SM-3MJGD |
|  |  | GTEX-PLZ5-1526-SM-3P5ZX |
|  |  | GTEX-OIZI-0126-SM-3NB13 |
|  |  | GTEX-YFCO-1726-SM-4W21S |
|  |  | GTEX-13OW6-0126-SM-5IJGM |
|  |  | GTEX-14A6H-2326-SM-5Q5B5 |
|  |  | GTEX-ZDTT-2026-SM-5K7TY |
|  |  | GTEX-P4QR-0226-SM-3NMCH |
|  |  | GTEX-1399Q-2826-SM-5IJEZ |

**Table S2.** **Accession numbers used for mouse RNA-seq analysis.**

| **BioProject** | **BioSample** | **SRA** | **Run** |
| --- | --- | --- | --- |
| PRJNA268132 | SAMN03256018 | SRX824009 | SRR1734775 |
|  | SAMN03256020 | SRX824007 | SRR1734773 |
|  | SAMN03256021 | SRX824008 | SRR1734774 |
|  | SAMN03256022 | SRX824006 | SRR1734772 |
|  | SAMN03256023 | SRX824011 | SRR1734777 |
|  | SAMN03256024 | SRX824012 | SRR1734778 |
|  | SAMN03256025 | SRX824013 | SRR1734779 |
|  | SAMN03256027 | SRX824010 | SRR1734776 |
|  | SAMN03256029 | SRX824015 | SRR1734781 |
|  | SAMN03256030 | SRX824016 | SRR1734782 |
|  | SAMN03256031 | SRX824017 | SRR1734783 |
|  | SAMN03256033 | SRX824014 | SRR1734780 |

**Table S3. Inserted annotation for *Tmprss6-2* and *Tmprss6-X2* RNA-seq analysis.**

| ***Tmprss6-2* (NM_001355601.1)** |
| --- |
| chr15 refseq exon 78439673 78440372 . - . transcript_id "NM_001355601.1"; gene_id "ENSMUSG00000016942"; gene_name "Tmprss6";  chr15 refseq exon 78440608 78440744 . - . transcript_id "NM_001355601.1"; gene_id "ENSMUSG00000016942"; gene_name "Tmprss6";  chr15 refseq exon 78442282 78442553 . - . transcript_id "NM_001355601.1"; gene_id "ENSMUSG00000016942"; gene_name "Tmprss6";  chr15 refseq exon 78443677 78443845 . - . transcript_id "NM_001355601.1"; gene_id "ENSMUSG00000016942"; gene_name "Tmprss6";  chr15 refseq exon 78444096 78444212 . - . transcript_id "NM_001355601.1"; gene_id "ENSMUSG00000016942"; gene_name "Tmprss6";  chr15 refseq exon 78445284 78445397 . - . transcript_id "NM_001355601.1"; gene_id "ENSMUSG00000016942"; gene_name "Tmprss6";  chr15 refseq exon 78446204 78446302 . - . transcript_id "NM_001355601.1"; gene_id "ENSMUSG00000016942"; gene_name "Tmprss6";  chr15 refseq exon 78446663 78446808 . - . transcript_id "NM_001355601.1"; gene_id "ENSMUSG00000016942"; gene_name "Tmprss6";  chr15 refseq exon 78452476 78452585 . - . transcript_id "NM_001355601.1"; gene_id "ENSMUSG00000016942"; gene_name "Tmprss6";  chr15 refseq exon 78452792 78452904 . - . transcript_id "NM_001355601.1"; gene_id "ENSMUSG00000016942"; gene_name "Tmprss6";  chr15 refseq exon 78454145 78454281 . - . transcript_id "NM_001355601.1"; gene_id "ENSMUSG00000016942"; gene_name "Tmprss6";  chr15 refseq exon 78454914 78455118 . - . transcript_id "NM_001355601.1"; gene_id "ENSMUSG00000016942"; gene_name "Tmprss6";  chr15 refseq exon 78459430 78459471 . - . transcript_id "NM_001355601.1"; gene_id "ENSMUSG00000016942"; gene_name "Tmprss6";  chr15 refseq exon 78459745 78459923 . - . transcript_id "NM_001355601.1"; gene_id "ENSMUSG00000016942"; gene_name "Tmprss6";  chr15 refseq exon 78460440 78460507 . - . transcript_id "NM_001355601.1"; gene_id "ENSMUSG00000016942"; gene_name "Tmprss6";  chr15 refseq exon 78461245 78461378 . - . transcript_id "NM_001355601.1"; gene_id "ENSMUSG00000016942"; gene_name "Tmprss6";  chr15 refseq exon 78465109 78465308 . - . transcript_id "NM_001355601.1"; gene_id "ENSMUSG00000016942"; gene_name "Tmprss6";  chr15 refseq exon 78468426 78469183 . - . transcript_id "NM_001355601.1"; gene_id "ENSMUSG00000016942"; gene_name "Tmprss6"; |
| ***Tmprss6-X2* (XM_006521417.2)** |
| chr15 refseq exon 78439673 78440372 . - . transcript_id "XM_006521417.2"; gene_id "ENSMUSG00000016942"; gene_name "Tmprss6";  chr15 refseq exon 78440608 78440744 . - . transcript_id "XM_006521417.2"; gene_id "ENSMUSG00000016942"; gene_name "Tmprss6";  chr15 refseq exon 78442282 78442553 . - . transcript_id "XM_006521417.2"; gene_id "ENSMUSG00000016942"; gene_name "Tmprss6";  chr15 refseq exon 78443677 78443845 . - . transcript_id "XM_006521417.2"; gene_id "ENSMUSG00000016942"; gene_name "Tmprss6";  chr15 refseq exon 78444096 78444212 . - . transcript_id "XM_006521417.2"; gene_id "ENSMUSG00000016942"; gene_name "Tmprss6";  chr15 refseq exon 78445284 78445397 . - . transcript_id "XM_006521417.2"; gene_id "ENSMUSG00000016942"; gene_name "Tmprss6";  chr15 refseq exon 78446204 78446302 . - . transcript_id "XM_006521417.2"; gene_id "ENSMUSG00000016942"; gene_name "Tmprss6";  chr15 refseq exon 78446663 78446808 . - . transcript_id "XM_006521417.2"; gene_id "ENSMUSG00000016942"; gene_name "Tmprss6";  chr15 refseq exon 78452476 78452585 . - . transcript_id "XM_006521417.2"; gene_id "ENSMUSG00000016942"; gene_name "Tmprss6";  chr15 refseq exon 78452792 78452904 . - . transcript_id "XM_006521417.2"; gene_id "ENSMUSG00000016942"; gene_name "Tmprss6";  chr15 refseq exon 78454145 78454281 . - . transcript_id "XM_006521417.2"; gene_id "ENSMUSG00000016942"; gene_name "Tmprss6";  chr15 refseq exon 78454914 78455118 . - . transcript_id "XM_006521417.2"; gene_id "ENSMUSG00000016942"; gene_name "Tmprss6";  chr15 refseq exon 78459745 78459923 . - . transcript_id "XM_006521417.2"; gene_id "ENSMUSG00000016942"; gene_name "Tmprss6";  chr15 refseq exon 78460440 78460507 . - . transcript_id "XM_006521417.2"; gene_id "ENSMUSG00000016942"; gene_name "Tmprss6";  chr15 refseq exon 78461245 78461378 . - . transcript_id "XM_006521417.2"; gene_id "ENSMUSG00000016942"; gene_name "Tmprss6";  chr15 refseq exon 78465109 78465343 . - . transcript_id "XM_006521417.2"; gene_id "ENSMUSG00000016942"; gene_name "Tmprss6";  chr15 refseq exon 78468426 78469183 . - . transcript_id "XM_006521417.2"; gene_id "ENSMUSG00000016942"; gene_name "Tmprss6"; |

**Table S4. Primers used for human TMPRSS6 isoforms detection using RT-PCR.**

| **Primer pair** | **5’-3’ Forward** | **5’-3’ Reverse** |
| --- | --- | --- |
| 1 | TGTTACTCTTCCACTCCAAAAGG | TAGATTCCCGGCGGGTAAGA |
| 1/2 | ATGCCCGTGGCCGAG | GTGCTCGGGGATTTGGAGAA |
| 3 | TGCCCTCTCTGGACTACGG | CTTGCTGCCCCTCTGAGATTG |
| 4 | ACAGCCATGACTACGACGTG | AGCAACATGTCTCTGAGCCT |

**Table S5. Primers used for mice *Tmprss6* isoform detection using RT-PCR.**

| **Primer pair** | **5’-3’ Forward** | **5’-3’ Reverse** |
| --- | --- | --- |
| Isoform 1 | TTCCAGCTCCCCTGTTCTACCA | CCAGCGTGGAATTCAGTACGACTAT |
| Isoform 2 | CCACTCTTGAAGGATGCCCACC | CAACAGTGGCGTGAAGCGGA |
| Isoform X2 | CGACTGACCCTGAGCCCTGA | GCGATAACAGCCCAGGATCACCA |

**Table S6.** **Primers and fragments used for cloning.**

| **TMPRSS6**  **construct** | **5’- 3’ Forward** | **5’- 3’ Reverse** |
| --- | --- | --- |
| TMPRSS6-2  TMPRSS6-3 (aa 9-461) | TTAAACTTAAGCTTGGTACCATGCCCGTGGCCGAGGCCCC | GGGGCCTCGGCCACGGGCATGGTACCAAGCTTAAGTTTAA |

**Table S7. gBlock fragments used for cloning.**

| **gBlocks** | **5’- 3’ Forward** |
| --- | --- |
| TMPRSS6-3 (aa 1-461) | TGGCTAGCGTTTAAACTTAAGCTTGGTACCATGTTGTTACTCTTCCACTCCAAAAGGATGCCCGTGGCCGAGGCCCCCCAGGTGGCTGGCGGGCAGGGGGACGGAGGTGATGGCGAGGAAGCGGAGCCAGAGGGGATGTTCAAGGCCTGTGAGGACTCCAAGAGAAAAGCCCGGGGCTACCTCCGCCTGGTGCCCCTGTTTGTGCTGCTGGCCCTGCTCGTGCTGGCTTCGGCGGGGGTGCTACTCTGGTATTTCCTAGGGTACAAGGCGGAGGTGATGGTCAGCCAGGTGTACTCAGGCAGTCTGCGTGTACTCAATCGCCACTTCTCCCAGGATCTTACCCGCCGGGAATCTAGTGCCTTCCGCAGTGAAACCGCCAAAGCCCAGAAGATGCTCAAGGAGCTCATCACCAGCACCCGCCTGGGAACTTACTACAACTCCAGCTCCGTCTATTCCTTTGGGGAGGGACCCCTCACCTGCTTCTTCTGGTTCATTCTCCAAATCCCCGAGCACCGCCGGCTGATGCTGAGCCCCGAGGTGGTGCAGGCACTGCTGGTGGAGGAGCTGCTGTCCACAGTCAACAGCTCGGCTGCCGTCCCCTACAGGGCCGAGTACGAAGTGGACCCCGAGGGCCTAGTGATCCTGGAAGCCAGTGTGAAAGACATAGCTGCATTGAATTCCACGCTGGGTTGTTACCGCTACAGCTACGTGGGCCAGGGCCAGGTCCTCCGGCTGAAGGGGCCTGACCACCTGGCCTCCAGCTGCCTGTGGCACCTGCAGGGCCCCAAGGACCTCATGCTCAAACTCCGGCTGGAGTGGACGCTGGCAGAGTGCCGGGACCGACTGGCCATGTATGACGTGGCCGGGCCCCTGGAGAAGAGGCTCATCACCTCGGTGTACGGCTGCAGCCGCCAGGAGCCCGTGGTGGAGGTTCTGGCGTCGGGGGCCATCATGGCGGTCGTCTGGAAGAAGGGCCTGCACAGCTACTACGACCCCTTCGTGCTCTCCGTGCAGCCGGTGGTCTTCCAGGCCTGTGAAGTGAACCTGACGCTGGACAACAGGCTCGACTCCCAGGGCGTCCTCAGCACCCCGTACTTCCCCAGCTACTACTCGCCCCAAACCCACTGCTCCTGGCACCTCACGGTGCCCTCTCTGGACTACGGCTTGGCCCTCTGGTTTGATGCCTATGCACTGAGGAGGCAGAAGTATGATTTGCCGTGCACCCAGGGCCAGTGGACGATCCAGAACAGGAGGTACCACTTCCTCTCCTCCCTCTGGCTTCCTTTCCTCCCTCCCCCTCCCTCTCTTCCCTCCTCAATAGTGACCCCCTCATTGGAAGCCCAAGTCCCCAATCTCAGAGGGGCAGCAAGGGGAGCGAGCAGAGGCTGGGGCTGGTGTCAGGCCTGTTGCCCTCTCGAGTCTAGAGGGCCCTTCGAAGGTAAG |
| TMPRSS6-4 (insertion) | TTACGGGCTGGGGCGCCTTGCGCGAGGGCGCCCTACGGGCGGATGCTGTGGCCCTATTTTATGGATGGAGAAACCAAGGCTCAGAGACATGTTGCTGCCCCATCAGCAACGCTCTGCAGAAAGTG |

**Supplemental figures**


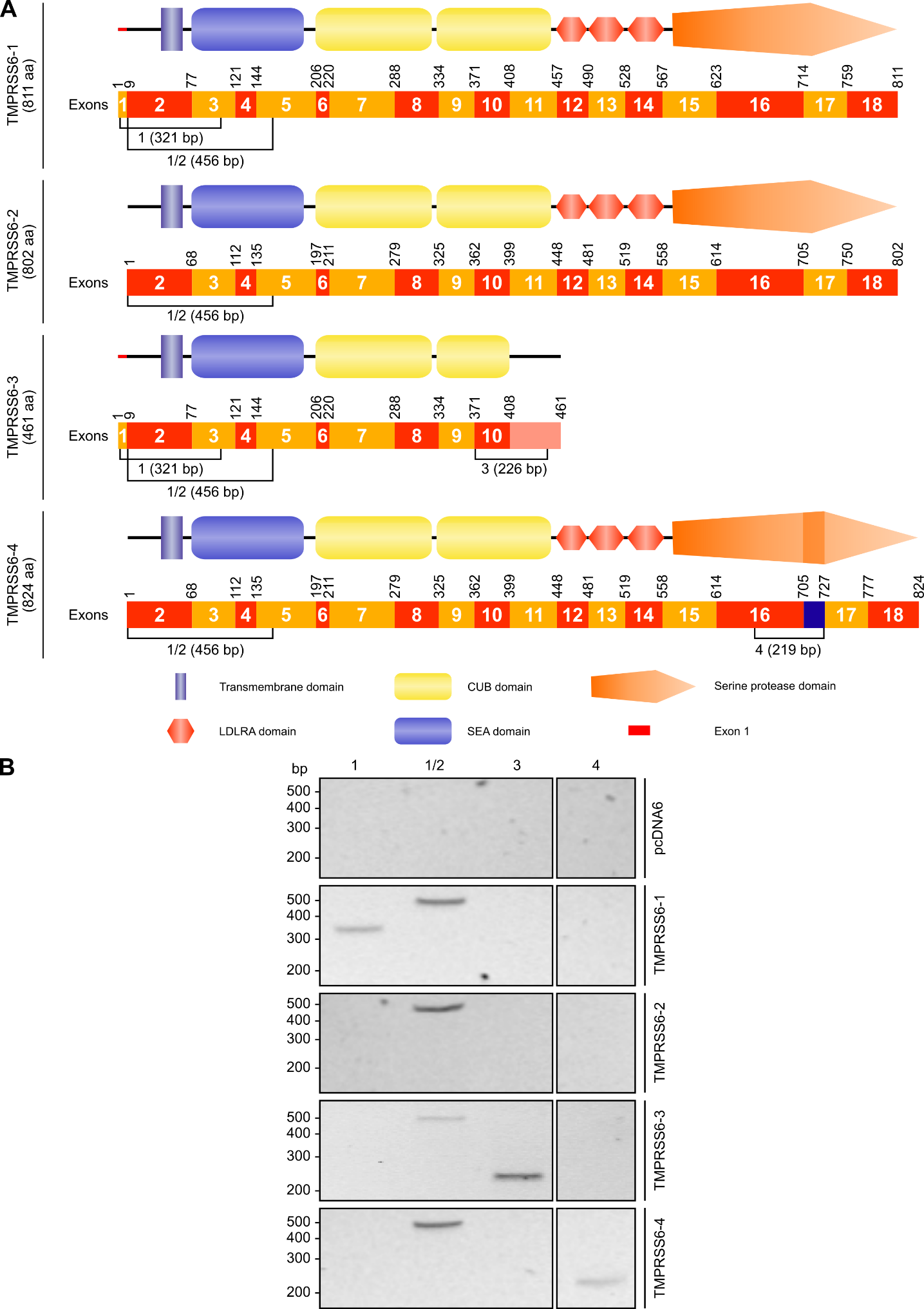


**Figure S1. Primers used for *TMPRSS6* isoforms detection by RT-PCR.** (A) Schematic representation of primers. (B) RT-PCR amplification on plasmid DNA.


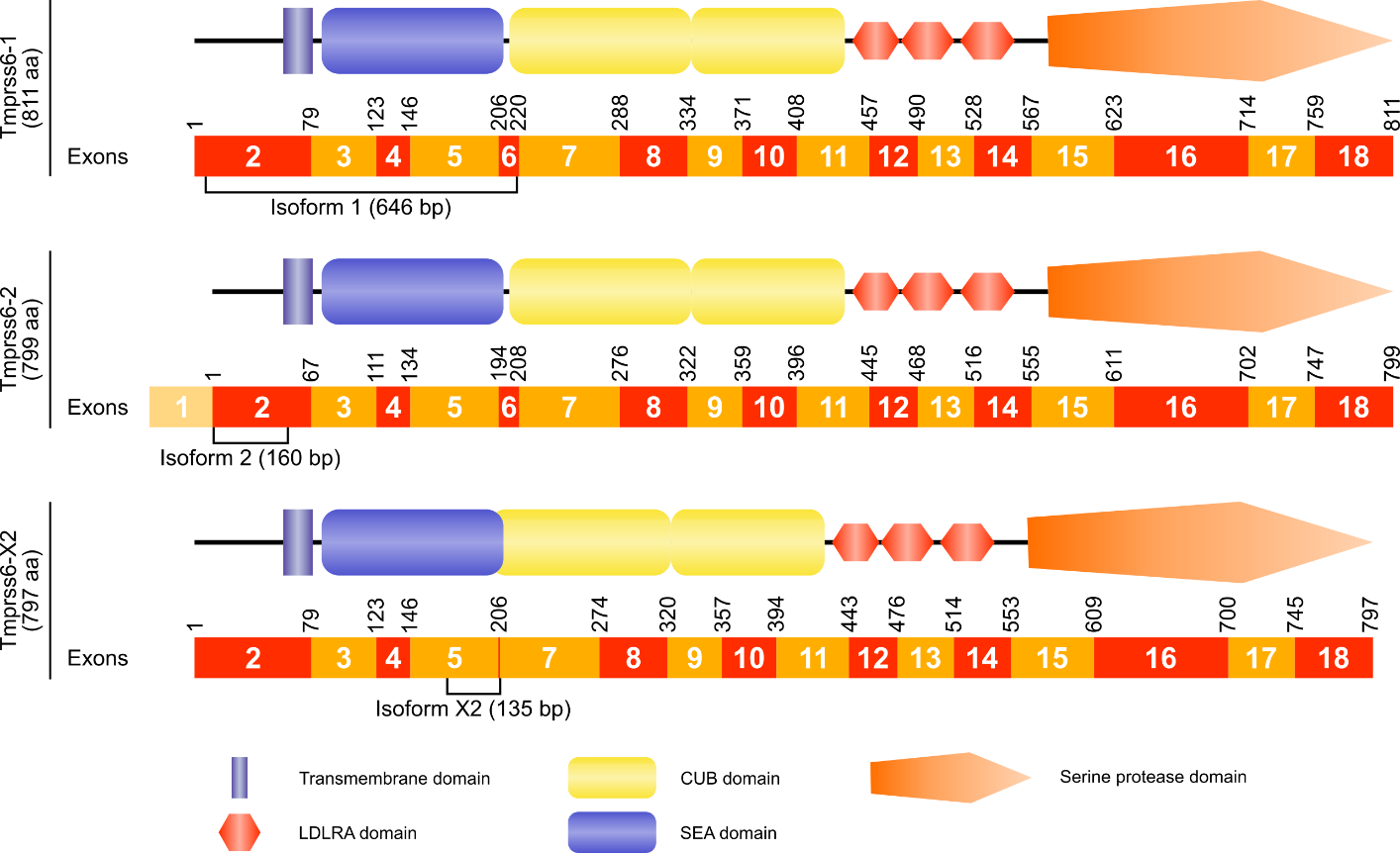


**Figure S2. Primers used for mice *Tmprss6* detection by RT-PCR.**


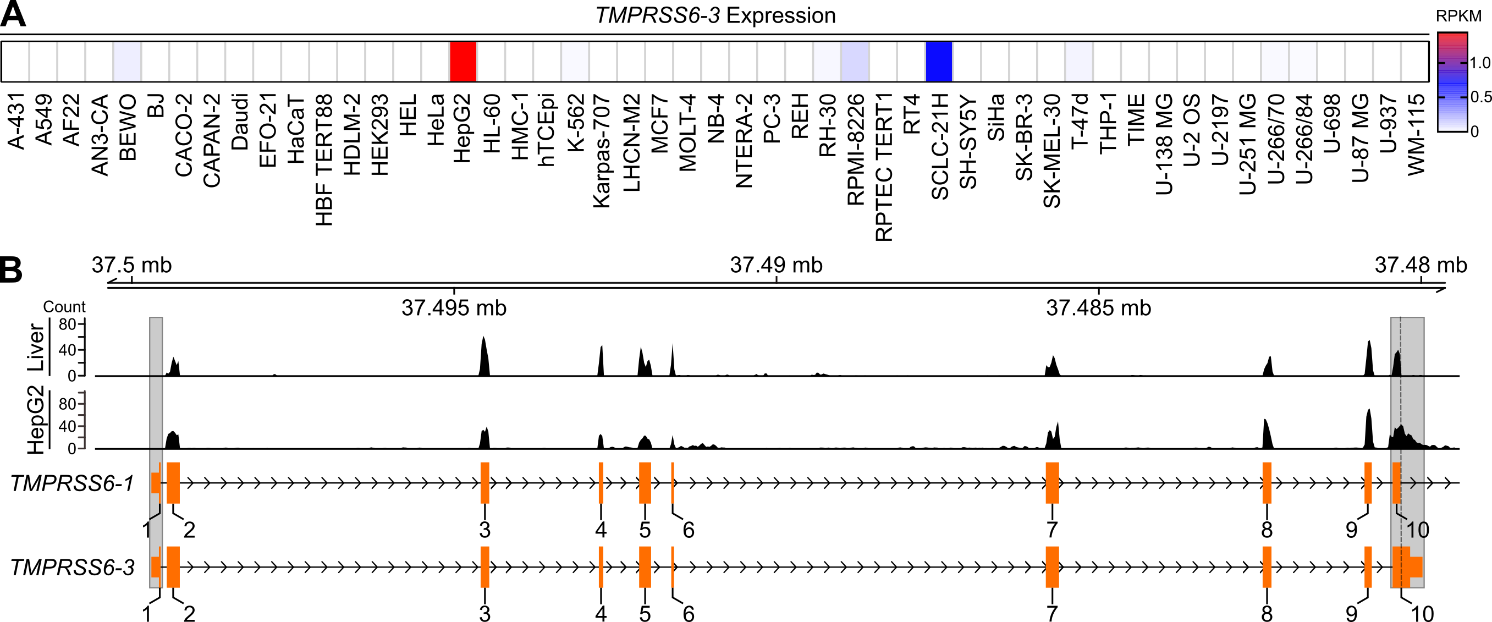


**Figure S3.** ***TMPRSS6-3* expression in human cell lines.** (A) *TMPRSS6-3* expression levels in human cell lines. Results are presented as a heat-map of RPKM determined by RNA-sequencing data analysis available from The Human Protein Atlas Project (*n* = 2). (B) Reads alignment of liver (Gene Expression Omnibus (GEO) accession GSM781695) and HepG2 (GEO GSM2400156) samples on TMPRSS6 gene (chromosome 22, human GRCh37 genome). Grey boxes represent exon 1 and exon 10. Non-coding regions are displayed smaller than coding regions.


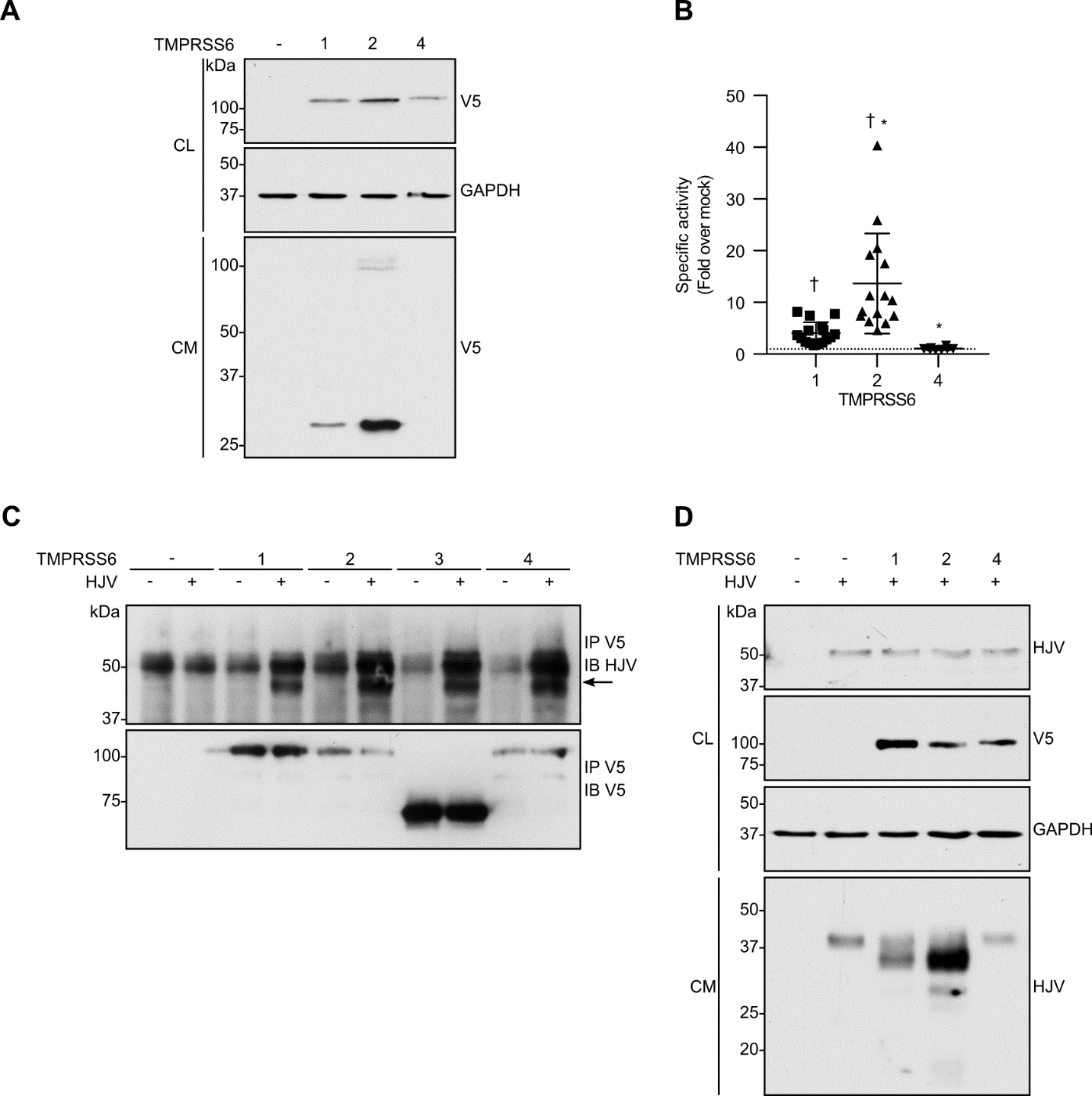


**Figure S4.** **TMPRSS6 isoform functionalities in HEK293 transfected cells.** (A) Cells were transfected with TMPRSS6-V5 isoforms 1, 2 or 4. Expression was detected by immunoblotting with an anti-V5-HRP antibody. Equal amounts of cell lysate (CL) and concentrated cell medium (CM) were loaded on 12% SDS-polyacrylamide gels. Cell lysate GAPDH was blotted as a loading control (n≥3). (B) Proteolytic activity was measured in the cell medium of cells transfected with TMPRSS6-V5 isoforms 1, 2 or 4. The fluorescence released by the cleavage of Boc-QAR-AMC (200 µM) was monitored. Results are presented as specific activity (fluorescence units/µl/µg of total proteins), are baseline corrected and are shown as scatter plot ± SD (n≥10). One-sample t test was used to analyze the activity of isoforms compared to mock. *P* values <.05 were considered statistically significant (†). Kruskal-Wallis test was used to compare the activity between the isoforms. *P* values <.05 were considered statistically significant (*). (C) Cells were co-transfected with TMPRSS6-V5-1, 2 or 4 and hemojuvelin (HJV). Immunoprecipitation was performed in cell lysates using an anti-V5 antibody. Samples were loaded on 12% SDS-polyacrylamide gels and immunoblotting was performed using anti-HJV or anti-V5 antibodies (n≥3). (D) Cells were co-transfected with TMPRSS6-V5-1, 2 or 4 and hemojuvelin (HJV). HJV cleavage in cell media was detected by immunoblotting with anti-HJV antibody. Equal amounts of cell lysate (CL) and concentrated cell medium (CM) were loaded on 12% SDS-polyacrylamide gels. Cell lysate GAPDH was blotted as a loading control (n≥3).
